# Potato Cultivar Identification in South Africa Using a Custom SNP Panel

**DOI:** 10.3390/plants11121546

**Published:** 2022-06-10

**Authors:** Inge Gazendam, Pinkie Mojapelo, Michael W. Bairu

**Affiliations:** 1 Agricultural Research Council-Vegetable, Industrial and Medicinal Plants (ARC-VIMP), Roodeplaat, Pretoria 0001, South Africa; mojapelop@arc.agric.za (P.M.); bairum@arc.agric.za (M.W.B.); 2 Faculty of Natural & Agricultural Sciences, School of Agricultural Sciences, Food Security and Safety Niche Area, North-West University, Private Bag X2046, Mmabatho 2735, South Africa

**Keywords:** potato fingerprinting, single nucleotide polymorphism, SNP panel, SeqSNP, KASP, SNP genotype database

## Abstract

DNA fingerprinting is a molecular technique applied to identify genetic differences between plant cultivars or lines and is used for genetic purity testing. The suitability of single nucleotide polymorphism (SNP) panels for the fingerprinting of tetraploid potato were investigated as a new high throughput, objective, and cost-effective method instead of simple sequence repeats (SSRs) and polyacrylamide gel electrophoresis (PAGE). One-hundred and ninety (190) potato cultivars, including various cultivars currently important in South Africa, were genotyped at 500 SNP positions utilising SeqSNP by LGC Biosearch Technologies. An optimal panel of 25 SNP markers was identified that could discriminate between South African potato cultivars on genetic allele dosage. The genotypes of these SNPs were validated on selected potato genotypes using KASP (Kompetitive Allele Specific PCR) SNP assays. A database of SNP genotype profiles was compiled for all the entries of the germplasm database. The panel of 21 successful SNPs accurately identified the unique potato cultivars in the database. The KASP SNP assays of the successful SNP panel are therefore available for potato DNA fingerprinting as new germplasm, or purity test requests are submitted to ARC-VIMP. This panel provides an objective method for assigning putative cultivar identity to unknown samples submitted for fingerprinting.

## 1. Introduction

DNA fingerprinting is a molecular technique applied to identify genetic differences between cultivars or lines and is used for genetic purity testing.

The benefit of potato (*Solanum tuberosum*) DNA fingerprinting is that it can be done at very early stages of development, such as mini-tuber or in vitro leaf material, and it is less resource-intensive than morphological methods. Accidental mixes can therefore be identified early, before in vitro multiplication, to prevent costly mistakes later on. Cultivar genetic identity is important in the protection of plant breeders’ rights.

The South African potato industry is the main client for which the Agricultural Research Council (ARC) provides genetic fingerprinting services. It is the largest vegetable commodity in South Africa, with a gross production value of R8.08 billion in 2019 [1]. The South African Seed Potato Certification scheme certified about 6.8 million (25 kg) bags of seed potatoes during the 2017/2018 production season [2]. Seed potato growers must maintain their cultivars’ genetic purity to provide true-to-type cultivars for the potato production industry. The in vitro gene bank of the ARC-VIMP is also dependent on the fingerprinting service and cannot release material to the industry unless trueness-to-type is confirmed. In this sense, DNA fingerprinting of potato cultivars benefits everyone involved in the industry. The fingerprinting of newly introduced lines during the potato certification process is definitely essential but is not yet mandatory [3,4]. All commercial varieties are tetraploid with 2n = 4x = 48 chromosomes.

Simple sequence repeat (SSR) fingerprinting is a popular method used for potato cultivar identification [5,6,7,8] and the evaluation of genetic diversity [9]. SSR markers have high mutation rates (due to DNA polymerase slippage during DNA replication) and provide high allele numbers per marker. Polymerase Chain Reaction (PCR) amplification of SSR sequences produces a mixture of DNA fragments from each DNA sample being compared. As a result of electrophoresis in a gel matrix, fragments are separated by size, resulting in a characteristic pattern (fingerprint) of bands from each sample. Differences are observed as the presence or absence of a particular fragment. If the fingerprint patterns of two plants differ, the plants are either not identical cultivars or their clones are not true-to-type. Five SSR markers have been used to effectively distinguish all South African cultivars at the ARC-VIMP until now [5]. SSR analysis, however, has some limitations related to throughput, cost, and the scoring of multiple alleles or stutter bands. Due to the indirect method of determining fragment sizes, the SSR allele database would not contain fragments of identical sizes, making cultivar comparisons difficult. 

Converting to the most modern method of fingerprinting currently available, namely SNP genotyping, was proposed. Due to advances in technology, it has become easier and cheaper to assay SNPs than ever before. Several authors suggest panels of SNP markers to replace SSR marker analysis for cultivar identification due to technical and economic reasons [10,11,12,13]. An SNP is the variation in a single nucleotide that occurs at a specific position in the genome of any organism. To be considered an SNP, the variation must be present in more than 1% of the population. If the variation is present at a lower frequency, it is considered a rare mutation (abnormal change). SNPs are highly abundant in plants and spread out evenly over the genome. Potato genomes are highly polymorphic, with one SNP occurring every 20 base pairs (bp). SNPs have been identified in significant quantities for potatoes using various high-throughput sequencing technologies [10,14,15]. These SNPs have a fixed location on the reference genome of potatoes [16] and are publicly available. They can be used in several types of flexible genotyping platforms, such as the KASP (Kompetitive Allele Specific PCR) genotyping platform (https://www.biosearchtech.com (accessed on 1 August 2019); [17]) or the development of SNP arrays [18]. The Infinium SolCAP 12 K array has been successfully utilised to fingerprint and characterise diverse potato collections of the Northwest Potato Variety Development program in the United States [19], EEA INTA Balcarce in Argentina [20] and the potato collection at the International Potato Centre (CIP) in Lima, Peru [21]. 

SNP markers are able to estimate allele dosage; thus, nucleotide genotype and copy number can be determined from a polyploid genome [12,13]. Copy number determination is impossible or produces highly variable results when SSR fragments are analysed.

Single-tube assays such as KASP (LGC Biosearch Technologies, Hoddesdon, United Kingdom) eliminate all post-PCR sample handling, thereby reducing the cost and time of SNP genotyping while lowering error rates [22]. Researchers found KASP to be a cost-effective and scalable SNP genotyping solution for small to moderate numbers of markers such as DNA fingerprinting for quality control analysis [23,24,25]. SNP calling is robust and accurate since specific primers and probe combinations bind to their complementary sites in the potato genome.

Identifying a small custom SNP panel that will be used to distinguish important potato cultivars in South Africa requires the screening of a larger number of SNPs. The SeqSNP^TM^ technique (LGC Biosearch Technologies) was chosen since it is more cost-effective for genotyping medium to large numbers of samples, making it cheaper for a greater number of SNPs than KASP SNP assays. SeqSNP is a targeted genotyping by sequencing (GBS) approach, which uses flexible, in-solution probe libraries to enrich targets before high-throughput sequencing [26]. Additionally, SeqSNP provides flanking sequence information for each SNP.

The aim of the study was to develop and optimise a protocol for migrating potato DNA fingerprinting from the old SSR and PAGE methods of fragment analysis to the most recent SNP genotyping method. 

## 2. Results

### 2.1. Assemble a Comprehensive Set of Commercial Cultivars Important to the South African Potato Industry for Developing the SNP Panel

A set of 190 tetraploid potato cultivars, including commercial cultivars important to the South African potato industry, were selected for developing the SNP panel. A list of the selected genotypes with reasons for their selection is presented in Appendix A, Table A1. 

### 2.2. SNP Data Mining and Identification of SNPs for Potato Genotyping

The aim of this step was to select a number of potato SNPs with high polymorphism information content (PIC) (with a minor allele frequency (MAF) close to 50%) from the literature to develop a small panel of SNPs that could be used to differentiate South African potato cultivars. 

The preliminary selection from the 14,530 successful SNPs [27] after filtering for the highest PIC values (33.6% < MAF < 50%) resulted in 1210 SNPs (results not presented). This represented an average marker interval from 0.42 Mb (Chr02) to 0.8 Mb (Chr12) per chromosome.

### 2.3. SeqSNP Genotyping of 190 Selected Potato Germplasms at 500 SNP Positions

SeqSNP is a targeted GBS approach with the added advantage of providing flanking sequence information of each SNP. The final selection of SNPs for SeqSNP after further filtering (as described in the Materials and Methods) was comprised of 500 SNPs, between 31 and 61 SNP markers per chromosome, corresponding to their size (Table 1). This represents an average marker interval of 1.40–1.46 Mb, respectively.

**Figure 1:**
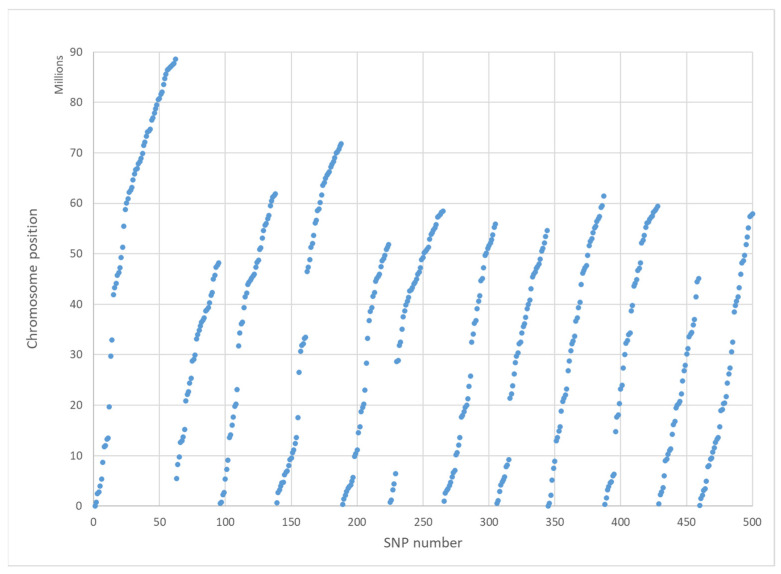
The positions of the final selection of 500 SNPs for SeqSNP, relative to the known potato genomic map.

The 500 SNPs’ positions relative to the known potato map are plotted in Figure 1 and indicate an even spacing of the selected SNPs over the 12 chromosomes. A low density of SNPs is characteristic around the centromeres of chromosomes since most non-coding SNPs were already removed.

The raw SeqSNP read number per sample ranged from 11 K to 1.2 million reads. 79–82% of reads per sample were retained after adapter clipping and quality trimming. The average effective target SNP coverage was 923-fold, much higher than what LGC Biosearch Technologies usually aim for (400-fold in tetraploids) (results not presented). One cultivar (“Connect”) yielded the most missing data, which were caused by lower than average read depth. Read depth was filtered to >100 reads before proceeding with the SeqSNP data analysis.

Of the 500 SNPs genotyped, 23 were not bi-allelic, i.e., two or more alternative alleles were observed. Among the 23, 6 had one or two occurrences of the third allele. There was only one monomorphic SNP among the 190 cultivars. In the three cultivars sent in duplicate (BP1, Mondial, and Up to Date), there were 28–30 SNP genotype differences per cultivar. These duplicate samples were from different sources and ages kept in the ARC-VIMP in vitro genebank (see Appendix A, Table A1).

The 500 SNPs were analysed for diversity in the genotyped population of 190 cultivars. REF (frequency of the reference allele) for the 500 SNPs ranged from 0.25 to 1. All the SNPs (except the lowest four) had PIC values between 0.22 and 0.5. Sixty-six percent (328 out of the 500) had REF values between 0.4 and 0.6, corresponding to 0.48 < PIC < 0.5.

**Figure 2:**
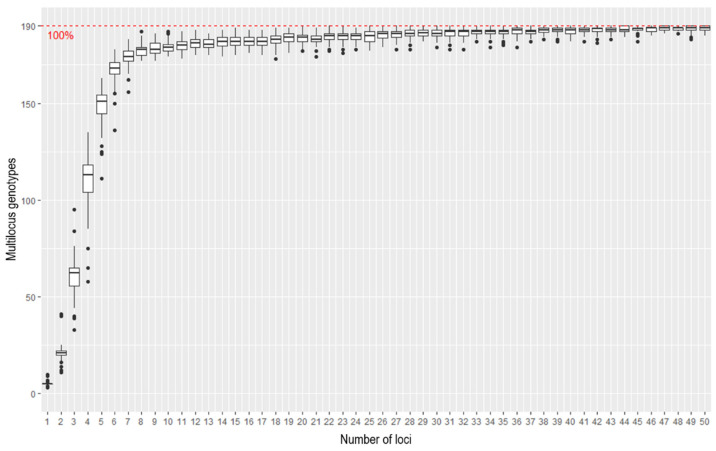
A genotype accumulation curve, used for determining the minimum number of loci necessary to discriminate between individuals in a population.

Genotype accumulation curves are useful for determining the minimum number of loci necessary to discriminate between individuals in a population. The function in R randomly samples loci without replacement and counts the number of observed multi-locus genotypes. According to the genotype accumulation curve (Figure 2), 185.73 out of 190 samples can be distinguished if only 25 loci are randomly chosen. With a number so close to 190, it is likely that a 25 SNP panel will be sufficient to discriminate genotypes and that a panel size of 25 is appropriate.

**Figure 3:**
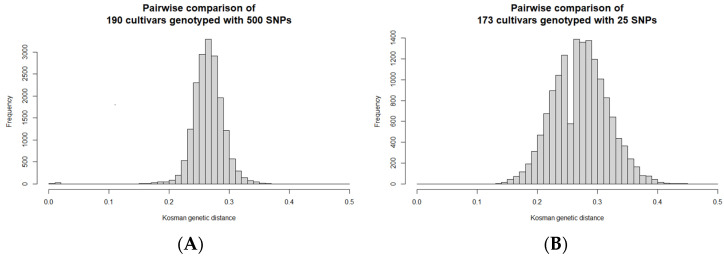
Histogram (frequency distribution) of pairs of potato cultivars (Y) vs. Kosman’s genetic distances (X). (**A**) 190 cultivars genotyped with SeqSNP at 500 SNP positions. Average distance = 0.265 ± 0.025. (**B**) 173 unique potato cultivars genotyped with SeqSNP at a selected panel of 25 SNPs. Average distance = 0.274 ± 0.044.

The pairwise genetic distances between cultivars were calculated (using Kosman’s index) to indicate the genetic relationships. The largest similarities are expected for similar or identical cultivars, as expected between the three duplicated pairs. Based on the Kosman genetic distances, 190 cultivars had 20 additional pairs of similar cultivars (0.008 < D < 0.020), possibly because of identical parental genotypes, being mixed, or having been mislabelled during in vitro maintenance or field planting. The genetic distances between the other unique pairs ranged from 0.154 to 0.374, with a mean of 0.265 ± 0.025 (Figure 3A). A dendrogram was constructed from the pairwise Kosman genetic distances to visually indicate the genetic relationships between the 190 cultivars genotyped at the 500 SNPs using SeqSNP (presented in Supplementary Figure S1).

### 2.4. Development of an Optimum Small SNP Panel to Discriminate Cultivars

The PIC and flanking sequence information obtained with SeqSNP was taken into account in selecting SNPs to be included in the KASP SNP assay design. The SNP diversity of the selected 25 SNP panel had REF ranging between 0.41 and 0.62 and all had PIC > 0.468, with 23 having PIC > 0.48. Distances between SNPs in the same linkage group were at least 0.58 Mb (results not presented). The identity of the SNPs and their chromosomal positions are restricted to protect the intellectual property right of the ARC to use the KASP SNP panel in the delivery of fingerprinting services. They were named alphabetically from A to Y.

One member of each similar pair and duplicated samples were removed to yield 173 distinct cultivars. The pairwise Kosman genetic distances calculated from the SeqSNP genotypes of these 173 cultivars genotyped with the 25 SNP panel indicates an upward shift in the genetic distances between the pairs, which ranged between 0.13 and 0.446, with an average of 0.274 ± 0.044 (Figure 3B). As a result, the panel is better at distinguishing cultivars based on genetic distance, despite using fewer SNP genotypes.

The ability of the selected panel to distinguish between unique cultivars (173 out of the 190) is demonstrated with a dendrogram of the pairwise genetic distances between cultivars using the 25 selected SNP panel (Supplementary Figure S2). When the final 25 SNP panel was selected, the 23 pairs of similar/identical cultivars (identified previously) differed by only two or fewer allele dosages. In the other pairwise comparisons, all cultivars differed by at least 10 allele dosages (D ≥ 0.13).

### 2.5. Validating of SeqSNP Genotyping Results with KASP SNP Assays of the Selected SNP Panel

Decisions regarding the most informative and best-performing SNPs needed to be made before an optimum panel of KASP SNP assays, able to discriminate between cultivars, could be ordered. LGC Biosearch Technologies cannot guarantee the success of the KASP SNP assay design or whether a successfully designed assay will produce a functional KASP SNP assay. The chosen SNP panel still needed to be validated by running real-time PCR experiments on each KASP SNP assay. The KASP SNP assays were ordered on 31 March 2021 and received on 6 July 2021.

**Figure 4:**
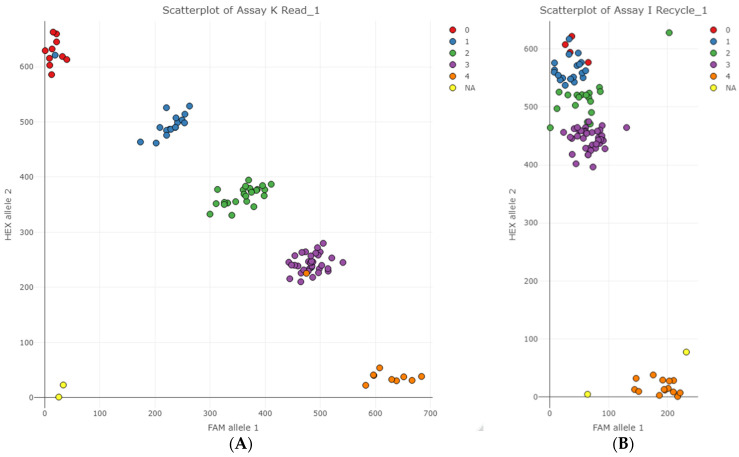
Examples of scatter plots constructed with KASP data points in the colour of the expected SeqSNP allele dosage. (**A**) An example of a successful KASP SNP assay, Marker K, able to cluster potato genotypes into the five gene dosage classes; (**B**) a scatter plot of one of the two unsuccessful KASP SNP assays, Marker I.

The 25 KASP SNP assays were validated on 78 selected potato germplasm (Supplementary Table S1), with three duplicated and additional germplasm selected per marker to represent all the expected genotypic classes. Figure 4A represents an ideal result in which KASP SNP assay values cluster into five distinct clusters. In this example of marker K, two genotypes did not cluster together as expected, as indicated by the coloured data points that do not match the rest of the cluster they grouped in.

Only two of the 25 KASP SNP assays failed to cluster into the five gene dosage classes (Figure 4B). In the case of marker I, allele 2 (HEX) competed with allele 1 (FAM) probably due to allele 2 primers’ preferential binding and amplification relative to allele 1. Conversely, Marker B exhibited preferential amplification of allele 1 (results not presented).

### 2.6. Calculate Genetic Relatedness between Cultivars and Draw Phylogenetic Trees to Indicate the Relationships between the Cultivars

**Figure 5:**
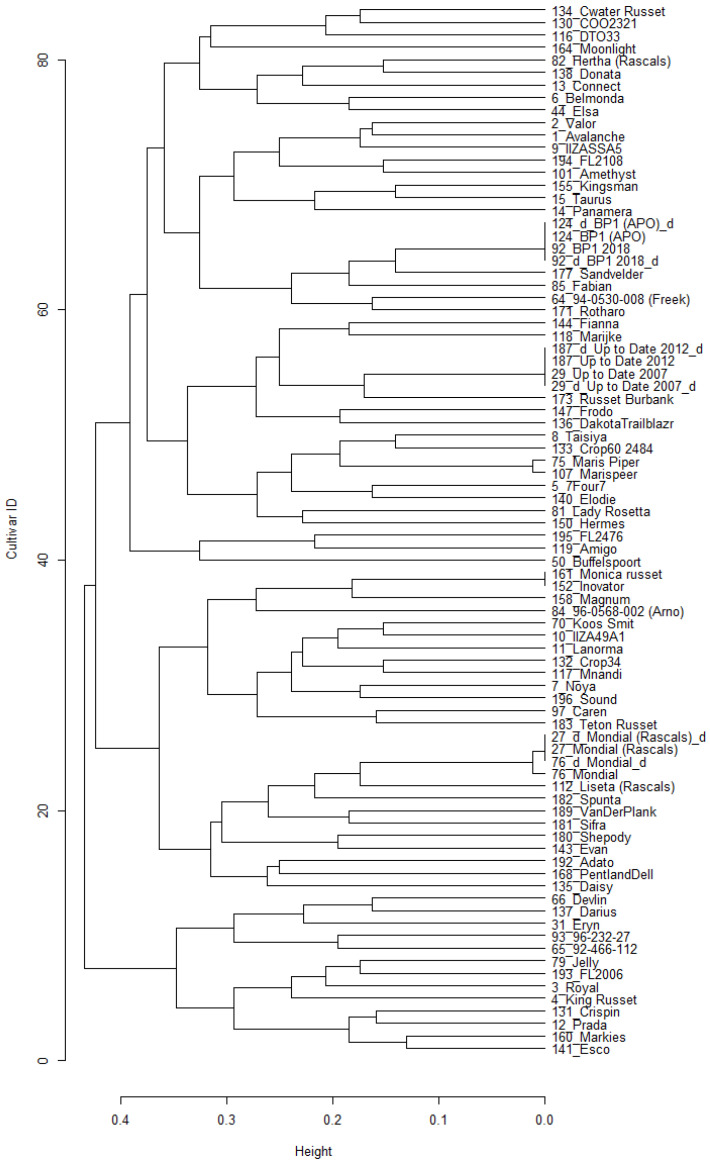
A complete cluster dendrogram of pairwise genetic distances of 78 selected commercially important cultivars genotyped with the 23 successful KASP SNP assays. Genetic distances were calculated with the Kosman index. Six cultivars were duplicated as indicated with “d” in their label.

Clustering of genotypes using Kosman’s index was done to demonstrate the ability of the 25 SNP panel to distinguish between cultivars. The largest similarities were expected between duplicated pairs. Since KASP markers B and I were unsuccessful, they were excluded from further analysis. The 78 samples that were genotyped with all the remaining 23 markers were selected. Cluster analysis using pairwise genetic distances (Figure 5) revealed that all cultivars could be distinguished from each other with at least one dosage difference, except in the following cases:

One sample of Mondial (sample 76) differed from the others (27, 27_d, 76_d) with one dosage at Marker E. Both fitPoly and the scatterplot results scored this sample as dosage 2, even though it was expected to be 1 like the others.

Maris Piper and Marispeer differed by only 1 dosage when compared with Marker M. These were members of the similar/identical pairs identified by SeqSNP genotyping using 500 SNPs. On the scatterplot of marker M, Maris Piper (sample 75) is one of the seven cultivars grouped in dosage class 3, while it was expected to be in dosage class 2. However, it was separated from the other dosage 3 class cultivars after increased cycle numbers (results not presented).

No distinction could be made with the 23 SNP panel between Innovator and Monica Russet. They had also previously been identified as members of the similar/identical pairs.

### 2.7. SNP Genotype Database

The germplasm SNP genotype database was set up with all the SeqSNP SNP genotypes and the KASP genotypes of samples assayed with 6 or more KASP SNP assays (Supplementary Table S2). It has already proven useful to assign putative cultivar identity to unknown samples submitted for fingerprinting by comparing their SNP genotypes to the germplasm SNP genotype database.

The obtained KASP results and expected SeqSNP genotypes were compared for the samples chosen for KASP verification. Approximately 96% of the genotypes obtained with the 23 successful KASP SNP assays corresponded to the genotypes expected with SeqSNP. Only 4% (88 out of 2139) of the reactions resulted in different genotypes, as scored by fitPoly. Samples that matched a different cultivar’s SeqSNP genotype were subsequently repeated and a few mixed DNA samples were identified (results not presented). 

Of the 23 successful KASP SNP assays, three had six or more mismatches between the expected SeqSNP genotype and obtained KASP genotype, each assayed with 93 samples; those were assay G with six, M with 17 and H with 26 mismatches. All four KASP samples of the duplicated Up to Date samples (29 and 187) differed from the SeqSNP results at marker G. For BP1 duplicates (92 and 124), both 124 KASP samples differed from the rest at marker H. 

After removing the two markers with high mismatch rates (H and M) from the panel, there were only 2.3% mismatches (45 out of 1953 samples) between KASP and SeqSNP genotypes.

### 2.8. Application of KASP SNP Assays

With this SNP panel, ARC-VIMP can provide fingerprinting services to clients in the potato industry.

A tool was developed to allow ARC-VIMP to select the smallest appropriate subset of markers to use for fingerprinting if a purity test request is received. Genotyping with markers that do not show any dosage difference is thereby avoided and fewer markers than the total panel are analysed, which is beneficial to the client. 

With the second tool, the ARC-VIMP can objectively assign putative cultivar identities to unknown samples submitted for fingerprinting by comparing their SNP genotypes with the germplasm SNP genotype database. The members in the database with the lowest pairwise genetic difference is identified. The probability that a random sample of potatoes in a population will have a particular DNA profile is dependent on the number of markers used and the allele frequency in the potato population. If we use more markers, or the rarer the allele frequencies are, the lower the match probability.

Table 2 illustrates the application of the product rule where the frequencies of the per-locus genotypes (or independent SNP markers in this case) are multiplied together to get the match probability. Random match probabilities are interpreted as the one in X chance of an unrelated cultivar having the same DNA profile as the unknown sample. Given the REF of each marker, there is a one in 87 chance that an unrelated cultivar will have the same DNA profile as “Example_cv” purely by chance.

## 3. Discussion

### 3.1. Assemble a Comprehensive Set of Commercial Cultivars Important to the South African Potato Industry for Developing the SNP Panel

A representative sample from a germplasm population is essential for developing or validating DNA assays. This step involved selecting a set of South African potato cultivars that represent all varieties relevant to developing a method applicable to South Africa. The 190 potato cultivars selected in this study could not contain all the important potato germplasms and is the minimum sample number for SeqSNP (LGC Biosearch Technologies). Higher numbers were not affordable with the funding available.

### 3.2. SNP Data Mining and Identification of SNPs for Potato Genotyping

As per [27], the average minor allele frequency (MAF) of SNP found in recently released potato varieties is about 10 times smaller than the average MAF of SNP found in varieties released before 1945. Thus, it is reasonable to conclude that new SNPs found in the recent potato varieties reveal a low allele frequency. The MAF value may also be used to determine the age of the allele. Allele frequencies of pre-1945 SNPs are relatively stable, and over a century of selective breeding did not affect them [27]. By filtering SNPs according to their high PIC values, we can select SNPs in the old founding cultivars, which might be able to differentiate between other sets of potato germplasm, such as South African potato cultivars and other cultivars selected in this project.

Major and minor allele frequencies are influenced by the specific population. For variety identification, it is more appropriate to refer to population allele frequencies relative to the potato reference genome (REF and ALT alleles) since it is more stable.

PIC is dependent on the allele frequency. A balanced allele frequency contributes to the highest discriminatory power, and the population allele frequency of SNPs affects the probability of each allele dosage. According to the definition given by Anderson et al. [28], PIC_i_ = 1 − ∑p^2^_ij_; were p_ij_ is the frequency of the allele j for each marker i. For bi-allelic SNP data, specifically, the formula can be rewritten as PIC = 1 − REF^2^ − (1 − REF)^2^, where REF is the frequency of the reference allele. Therefore, a PIC value of 0.5 corresponded to the theoretical maximum for bi-allelic markers. When REF closes to 50%, the PIC reaches the highest value (0.5).

### 3.3. SeqSNP Genotyping of 190 Selected Potato Germplasms at 500 SNP Positions

SeqSNP was initially planned to be performed on genomic DNA (gDNA) available in the freezer for many potato cultivars. The quality of the gDNA is, however, important for Next Generation Sequencing (NGS) analysis. The sampling kits from LGC Biosearch Technologies were therefore utilised and gDNA was isolated by them in a 96-well format in a strategy to improve the success of NGS during SeqSNP. The turnaround time for SeqSNP was one week after receiving the samples for gDNA extraction.

### 3.4. Development of an Optimum Small SNP Panel to Discriminate Cultivars

The application of SSR/SNP markers in crop improvement will depend on the quality of the information they provide regarding genetic diversity and population structure parameters. SNP fingerprinting provides both nucleotide genotype and copy number data for each allele, which is an advantage over SSR fingerprinting. SSR markers tend to have more alleles per locus than bi-allelic SNPs. Therefore, more SNPs (7–11 for maize per SSR) are needed to replace a single SSR. Due to their unique features, such as abundance in the genome and the ability to generate polymorphism at the single-base level, SNP markers are more cost-effective, technically feasible, and high throughput to measure. Ref. [12] proposed a panel size of 40–50 SNPs with a minor allele frequency between 40 and 60% for potatoes.

The population allele frequency affects the discriminatory power of SNP loci, where a balanced allele frequency (50%) yields a higher discriminatory power (maximum PIC = 0.5). However, selecting SNP loci with unbalanced allele frequencies (low PIC) can hardly identify differences between varieties (average Kosman similarity coefficients tend toward 1.00 as REF drops, according to Figure 2 in [12]). Another factor that affects SNP’s discriminatory power is the number of markers in the panel. It was previously found that a larger panel size (>50) did not significantly improve the pairwise comparisons’ average similarity value and variance [12]. However, a too-small panel may result in many similar pairs (Figure 3 in [12]). Despite this, a panel of 25 SNPs was proposed for this study due to cost concerns. 

For polyploid data, the Kosman similarity coefficient is superior to the Jaccard coefficient (for binary data, such as the presence/absence of an SSR allele) since it compares every allele dosage from different genotypes. It is, therefore, more sensitive to detect dissimilarity within a small SNP panel. As recommended by [12], a Kosman genetic similarity (1 − dissimilarity) of less than 0.85 fails to find a similar variety. Based on the chosen SNP panel, all unique cultivars were genetically distant from one another by at least 0.13 (Supplementary Figure S2).

Only tetraploid commercial cultivars (2n = 4x = 48) were considered in this study. Cultivars with ploidy other than tetraploid may generate imprecise Kosman genetic distances with the tetraploid cultivars.

### 3.5. Validating of SeqSNP Genotyping Results with KASP SNP Assays of the Selected SNP Panel

The two failed KASP markers (B and I) had no flanking variants that would have interfered with the assay. There was competition between one allele primer and the other, caused by preferential binding and amplification of one allele over the other. Therefore, these assays are not useful to genotype and classify potato cultivars into their dosage classes.

The original fitTetra tool only allows for the genotyping of autotetraploids and the clustering of SNP genotypes into five gene dosages [29,30]. In the meantime, an extension to higher levels of auto-polyploidy was implemented into a more advanced version of the package called fitPoly (https://cran.r-project.org/package=fitPoly (accessed on 26 May 2021)). This package was used to cluster and dose-call the tetraploid potato KASP genotyping results in R.

### 3.6. Calculate Genetic Relatedness between Cultivars and Draw Phylogenetic Trees to Indicate the Relationships between the Cultivars

The author from [12] suggested a Kosman’s coefficient of 0.85 as a threshold for discriminating between similar and different varieties. Genetic similarities (similarity = 1 − dissimilarity) between non-duplicated potato samples using the 23 successful KASP SNP assays were in all cases above 0.87 (Figure 5), indicating that the 78 selected cultivars could be distinguished from each other.

### 3.7. SNP Genotype Database

KASP and SeqSNP genotypes of the 190 potato samples were combined to generate the germplasm SNP genotype database (Supplementary Table S2).

Marker O was expected to be the only marker, based on the SeqSNP genotyping, to distinguish a single dosage difference between cultivars Monica Russet (161) and Innovator (152) (Supplementary Figure S3). However, when both genotypes were assessed with the KASP SNP assays, they showed the same dosage. Consequently, the KASP SNP panel failed to distinguish between these two cultivars. It is proposed that during SeqSNP the clustering of the NGS reads indicated a difference when they were in actual fact the same at this marker. Since KASP SNP assays will be used further during potato SNP fingerprinting, the genotypes obtained with the KASP method are the accepted ones.

Marker H resulted in 26 dosage differences from the expected SeqSNP genotypes and obtained KASP genotypes over the 93 samples assayed. Marker M had the second-highest number of mismatches at 17. The KASP SNP assays for these markers may be detecting a different SNP than the target assayed by SeqSNP. It is therefore suggested that these two markers be dropped and that the remaining 21 SNP panel be implemented for potato SNP fingerprinting. A dendrogram of pairwise genetic distances using the selected 21 SNP panel could still distinguish between all cultivars, except the previously detected similar or duplicated pairs (Supplementary Figure S3).

### 3.8. Application of KASP SNP Assays

Utilising the developed R Studio scripts, ARC-VIMP can generate scientifically significant SNP fingerprint profiles to distinguish a cultivar from a suspected case, or objectively assign putative cultivar identities to unknown samples submitted for fingerprinting by comparing their SNP genotypes with the germplasm SNP genotype database. 

For bi-allelic markers, such as the SNPs employed here, an REF value of 0.5 corresponds to the theoretical maximum, resulting in the highest polymorphism information content (PIC) of 0.5. SNP markers were specifically selected for this project to have a balanced allele frequency. All SNPs selected for the panel had REFs between 0.41 and 0.62 among 190 samples, and all PICs were above 0.46. The probability of 0 (zero) dosage differences between two samples, if REF is 0.5, is 0.27 [12]. Therefore, the probability of 0 dosage differences between two samples over 10 markers in potato is 0.27^10^, corresponding to one pair from a panel of 986 samples being indistinguishable by chance alone.

## 4. Materials and Methods

### 4.1. Assemble a Comprehensive Set of Commercial Cultivars Important to the South African Potato Industry for Developing the SNP Panel

A germplasm set was obtained from the ARC-VIMP in vitro gene bank cultivar or contract collections. Included on the list were all 10 potato varieties that were deemed important during the 2018/2019 growing season [2]: Mondial, Sifra, Lanorma, FL2108, Panamera, Valor, Markies, Innovator, Up-to-Date and Taurus. Due to the inaccessibility of all national cultivar collections and commercially important entries, suggestions and availability were considered, and some cultivars were revived from in vitro long storage. Additionally, commercial clients were invited to contribute and make requests. Twenty potato cultivars were received from different companies. Eleven cultivars (7Four7, Belmonda, Connect, IIZA49A1, IIZASSA5, King Russet, Lanorma, Noya, Prada, Royal, Taisiya) were obtained from GWK Trading, three (FL2006, FL2108, FL2476) from Pepsico (Mr Frank Ossler), five (Adato, Avalanche, Fianna, Markies and Sound) from First Potato Dynamics (FPD) (Mr Theuns van Rensburg) and two (Panamera, Taurus) from Rascal Seed Research Laboratories (Mr Dawie Ras). Additionally, McCain Foods (Ms I Vorster) requested the inclusion of 96-0568-002 (Arno), Amigo, Crop60, Clearwater Russet, Dakota Trailblazer, Magnum, Monica Russet, Royal and Teton Russet. 

The germplasm list was annotated as follows: 1 = important commercial cultivars according to Potatoes South Africa annual reports, 2 = on the South African potato variety list (Department of Agriculture, Land Reform and Rural Development (DALRRD)), 3 = if the cultivar has already been fingerprinted at the ARC-VIMP using SSRs, and 4 = cultivars overlapping with those used by [11,27] (Vos gt). 

Germplasm received as tubers were planted in a greenhouse on 17 August 2020 after being treated with Rindite (ethylene chlorhydrin—ethylene dichloride—carbon tetrachloride 7:3:1) to break the dormancy.

Many of the selected germplasm entries were sampled on 29 October 2020 as leaves from a field at Zeekoegat, Roodeplaat, Pretoria (GPS Coordinates 25°37′05.3″ S 28°19′19.2″ E), planted for cultivar characterisation. Leaf disks were punched and placed into the 96-well sample collection plate (BioArk, LGC Biosearch Technologies), the desiccant was applied and sealed in a plastic bag. For the remaining accessions, leaves were collected from in vitro plants in batches between 2 to 25 November 2020. The samples were processed into a second plate and frozen before being freeze-dried and packed with the desiccant. The sampling plates with dried leaf samples, accompanied by a description for customs, order documentation and plate map file, were sent by courier to LGC Biosearch Technologies GmbH, Berlin, Germany, on 26 November 2020.

### 4.2. SNP Data Mining and Identification of SNPs for Potato Genotyping

A set of 20,000 SNPs, obtained from [27], were filtered to select over 1000 SNPs for SeqSNP assay design. These 20,000 SNPs were used in a previous study to screen 569 genotypes representing commercial potato cultivars and advanced breeding lines from the Netherlands [27]. These SNPs were mostly derived from [10,14,15]. Of these 20,000, only the successful 14,530 SNPs were considered (the same approach used by [12]). The SNPs were filtered to be informative (high PIC value) and spread out evenly over all the 12 potato chromosomes. All non-coding SNPs were removed to minimise assay failure rates since coding regions had a lower assay failure rate than non-coding regions [11,27]. No chloroplast, unmapped SNPs or SNPs designed for resistance genes were selected. The top 1500 SNPs with minor allele frequency values higher than 33.6% were further filtered to remove SNPs denser than 0.1 Mb. Low-density chromosomal areas were identified by looking at plotted coordinates of SNPs. More SNPs were chosen in these areas and for chromosome 12, which had a low marker density. 

### 4.3. SeqSNP Genotyping of 190 Selected Potato Germplasms at 500 SNP Positions

The resulting list of 1210 sequences containing SNPs were compiled into a file format with specific headings (BED file) and sent to LGC Biosearch Technologies to design SeqSNP probe assays. LGC Biosearch Technologies analyzed the BED file list of 1210 SNPs’ coordinates to determine if they yield the expected SNP alleles according to the potato reference genome (ST4.03, http://spuddb.uga.edu/pgsc_download.shtml (accessed on 20 March 2020)) [31]. 

Of the 1210 selected SNP markers, 11 markers with unknown chromosome position were excluded and 1199 SNP assays were designed successfully by LGC Biosearch Technologies. Of the successful SNPs, 94% (1130) were covered with two oligo probes and had no off-target hits to the potato genome. 

After probe design, the list was reduced to 500 SNPs by filtering according to high specificity (no off-target hits allowed), primer annealing temperature inside the range of 45–60 °C, and primer Tm differences of the probes not more than 10 °C. Filtering for large MAF while considering spacing (according to the chromosome coordinate of the SNP) was done manually to ensure that the final selection of SNPs are not closely linked and span as much of the genome as possible. 

Genomic DNA extraction and genotyping using SeqSNP of the selected set of genotypes was done at LGC Biosearch Technologies. 75 bp single reads were generated on an Illumina NextSeq 500/550 v2 sequencer. The demultiplexing of libraries was performed using barcodes, reads were clipped for adapter barcodes and quality trimmed, and aligned against the potato reference genome (ST5149G_2) with Bowtie2 v2.2.3. Variant discovery and genotyping of samples were performed with Freebayes v1.2.0, a Bayesian variant caller that provides a most likely genotype [32]. The flanking variant environment was determined from variant calling performed by LGC Biosearch Technologies on the alignment of the raw SeqSNP sequencing reads. 

### 4.4. Development of an Optimum Small SNP Panel to Discriminate Cultivars

The SeqSNP data (VCF file) was successfully imported into the R software package and filtered for read depth and quality [33]. Allele frequencies were extracted with gt.to.popsum of the vcfR package in R [34]. The PIC value for each SNP was calculated using the formula PIC = 1 − REF^2^ − (1 − REF)^2^, where REF is the frequency of the reference allele. The genotype accumulation curves up to a maximum number of 100 loci were drawn in R with poppr v2.9.3 [35,36].

The Kosman genetic distance method was implemented to calculate the pairwise difference in genotypes between individuals. This method considers the allele dosage scores and averages the value over all loci [37]. gd.Kosman in the PopGenReport package in R [38] was used.

R and Microsoft Excel were used to determine the number of flanking variants and distance to each target SNP. Various parameters were considered, and an iterative software pipeline was developed to enable the selection of an optimal panel for discriminating among South African potato cultivars on the genetic allele dosage. SNPs were marked in the flanking sequences, and a maximum number of one SNP both upstream and downstream of the target SNP, but no SNP closer than 20 bases, was allowed.

### 4.5. Validating of SeqSNP Genotyping Results with KASP SNP Assays of the Selected SNP Panel

The completion of SeqSNP analysis enabled the procurement of an optimal panel of KASP SNP assays. With nearby SNPs marked in the flanking sequences, and the target SNP indicated with a “/” between the two nucleotides in square brackets [/], the sequences were submitted to LGC Biosearch Technologies on 19 March 2021 for their KASP assay design software. Primers were supplied without primer sequences but with specific assay codes to allow re-ordering.

Using the CTAB extraction method following standard laboratory protocols, genomic DNA (gDNA) was isolated from field-grown, in vitro, and producer-supplied potato lines for verification with KASP SNP assays. The leaf samples (100 mg) were ground in the Genogrinder (SpexSamplePrep) at 1500 rpm for 4 min before adding the CTAB isolation buffer [2% CTAB, 1.5 M NaCl, 20 mM EDTA, 0.1 M Tris-HCl, 0.2% β-mercaptoethanol]. A 30-min incubation period at 60 °C was followed by extraction with an equal volume of chloroform and isoamyl alcohol (24:1). The samples were centrifuged for 10 min at 10,000× *g* and the supernatant was transferred to a clean tube. Genomic DNA was precipitated with 0.6 volumes of isopropanol for 30 min at −20 °C. After centrifuging at 10,000× *g* for 10 min, the pellet was washed with 70% ethanol. The DNA pellet was air dried and resuspended in 1× TE buffer overnight, and DNA concentrations were determined with the Nanodrop ND-1000. Distilled water was used to prepare dilutions of 20 ng/μL.

KASP SNP assays were run according to the manufacturer’s manuals and recommendations [39,40,41]. 10 μL of KASP SNP assay reaction volumes consisting of 1× KASP genotyping master mix, 1× KASP probe mix, and 100 ng template gDNA per KASP SNP assay reaction were added to 96-well PCR plates (Biorad Hard-shell PCR plates 96-well, thin wall, clear well, HSP9601). Those cultivars that are important to the industry and represent all the allele dosage groups for each SNP marker were selected from the SeqSNP genotype dosage data (Supplementary Table S1). Plates were sealed with optically clear seals (Biorad Microseal “B” adhesive sealing film, MSB1001). Reactions were run on a Biorad CFX96 Connect real-time PCR machine. The cycling conditions were 15 min at 94 °C for hot-start Taq activation, a 2-step 65–57 °C touchdown protocol over 10 cycles, and 26 cycles after touchdown at 94 °C and 57 °C. The end-point fluorescence data for FAM and HEX were read at 30 °C.

R scripts were developed to analyse KASP SNP assay data. Scatter plots were constructed with the data points in the expected SeqSNP allele dosage colour. The software package in R called “fitPoly” [29,30] (https://cran.r-project.org/package=fitPoly (accessed on 26 May 2021)) was used for clustering and SNP dosage calling. It can fit the most accurate model for clustering polyploid genotyping data.

### 4.6. Calculate Genetic Relatedness between Cultivars and Draw Phylogenetic Trees to Indicate the Relationships between the Cultivars

Kosman’s index was used to calculate pairwise genetic distances between cultivars’ KASP genotypes [37,38]. Dendrograms were constructed from the allele dosage scores of the SNP markers via hierarchical cluster analysis of the pairwise Kosman genetic distances, using the hclust package in R and by using the complete clustering method [42]. The SNP markers were also combined in the appropriate panels for some comparative analysis.

### 4.7. SNP Genotype Database

Set-up of an SNP genotype database for all the entries in the germplasm database was done. All the commercial potato cultivars considered are tetraploid, so the reference allele dosage is represented by a number between 0 and 4. Both expected SeqSNP and obtained KASP dosages (only cultivars assayed with 6 or more KASP SNP assays) were included in the database (Supplementary Table S2).

### 4.8. Application of KASP SNP Assays

#### 4.8.1. Selection of an Appropriate Subset of Markers to Distinguish between a Set of Cultivars

A script was developed in R Studio (https://www.rstudio.com/products/rstudio/ (accessed 5 November 2018)) that calculates the differences in allele dosages between two cultivars of interest at each of the SNP markers in the panel. Both SeqSNP and KASP dosages were included, if available. The input is the names of two cultivars that need to be distinguished with DNA fingerprinting. The dosage differences per marker are calculated and then sorted from high to low. The output is a list of markers that will give the highest confidence in the results if a difference in genotype between two suspected cultivars is detected. 

#### 4.8.2. Assign Putative Cultivar Identity to Unknown Samples Submitted for Fingerprinting 

A second script was developed in R Studio which determines a query sample’s most likely cultivar identity after genotyping it with several KASP markers, by comparing it to the germplasm SNP genotype database. 

The input is the KASP dosage score of the query sample that needs to be identified. The input file is temporarily combined with the KASP and SeqSNP genotype databases. The pairwise Kosman genetic distances are then calculated and sorted, and the lowest pairwise genetic differences that contain the query sample are listed as possible matches.

The probability that a match to a particular multiple-locus genotype would occur by chance is calculated using the “product rule”, taking the specific marker and its REF into account. Per-locus genotype frequencies are multiplied together to determine the match probability. For a diploid organism, the addition of a factor of 2 for each heterozygous locus is also included. Tetraploids are calculated differently but follow the same rule. For tetraploid individuals, the expected frequencies of genotype classes in progeny after random mating can be mono-allelic for one allele (AAAA) Riiii = p_i_^4^, bi-allelic simplex (AAAB) Riiij = 4p_i_^3^p_j_, bi-allelic duplex (AABB) Riijj = 6p_i_^2^ p_j_^2^, bi-allelic triplex (ABBB) Rijjj = p_i_4p_j_^3^ and mono-allelic for the second allele (BBBB) Rjjjj = p_j_^4^. If the REF (p_i_) is 0.5, the probability of a variety having 0, 1, 2, 3, or 4 allele dosages can be computed as 0.0625, 0.25, 0.375, 0.25, and 0.0625, respectively. An example calculation of random match probabilities is presented in Table 2, showing the slight difference in values obtained when REF = 0.5 versus the actual REF of each marker is used. 

## 5. Conclusions

A panel of 25 SNPs were verified with KASP SNP assays for the fingerprinting of potato cultivars in the certification process of seed potatoes. The panel of 21 SNP markers, after eliminating the ineffective KASP SNP markers B, H, I, and M, is able to distinguish between all potato cultivars, except the previously detected similar or duplicated pairs.

The development of an SNP genotype database, for a large number of potato cultivars crucial to the South African potato industry, was one of the main outputs of this project. SNP genotypes simplify the germplasm genotype database and enable us to compare the genetic profile of the unknown cultivar to the databased genotypes to determine the suggested identity of the cultivar. 

The KASP SNP assays developed for the selected SNP panel are suitable for genotyping samples locally as new germplasm, clonal identification or purity test requests are submitted to the ARC-VIMP. DNA fingerprinting based on SNP technology streamlines the process. Compared to conventional SSR and PAGE, the new technology offer improved efficiency, reliability, sensitivity, higher throughput and lower cost per sample. This 21 SNP panel also provides an objective method of assigning putative cultivar identity to unknown samples submitted for fingerprinting.

The custom SNP panel for SNP fingerprinting was developed on a selection of only 190 potato cultivars. There exists, therefore, the possibility that a new cultivar cannot be distinguished from others by this panel. However, highly informative SNPs (PIC close to 0.5) were selected, which correspond to SNPs in the old founding cultivars (released before 1945 in the history of potato breeding [27]). This panel is therefore expected to be useful to discriminate between wider sets of potato germplasm, enabling the addition of new cultivars to the SNP genotype database.

## Figures and Tables

**Table 1:** The number of SNP markers per potato chromosome for SeqSNP of potato samples with 500 selected SNP markers. The average marker interval and minimum and maximum minor allele frequency (MAF) of selected SNPs (according to [27]) are also presented.

Chromosome	No. of Markers	Largest Coordinate (Mb)	Ave Marker Interval (Mb)	Minimum Minor Allele Frequency (MAF)	Maximum Minor Allele Frequency (MAF)
Chr 1	61	88.6	1.45	34.9	50.0
Chr 2	33	48.3	1.46	32.7	49.9
Chr 3	43	61.9	1.44	26.3	49,9
Chr 4	50	71.8	1.44	31.0	49.8
Chr 5	36	51.9	1.44	28.6	49.9
Chr 6	41	58.5	1.43	37.1	49.2
Chr 7	40	55.9	1.40	31.2	49.8
Chr 8	39	54.7	1.40	27.3	50.0
Chr 9	43	61.5	1.43	28.4	49.3
Chr 10	41	59.4	1.45	29.6	49.9
Chr 11	31	45.1	1.45	31.5	49.7
Chr 12	42	59.3	1.41	32.0	49.8
Total:	500	716 Mb			

**Table 2:** A calculation of the random match probability of a query sample after genotyping with a small number of SNP markers. This calculation illustrates the difference between using REF as 0.5 or as the genotype allele frequency as determined for 190 potato cultivars.

	Genotype: KASP Dosage per Marker					
**Sample**	**X**	**F**	**Y**	**O**		
Example_cv	2	2	2	3		
	**Genotype frequency per locus:**				**Match probability**	**Interpretation**
if REF = pi = 0.5	0.375	0.375	0.375	0.25	0.0132	one in 76
use specific allele frequency for marker	0.3495	0.3671	0.3318	0.2704	0.01151	one in 87

## Data Availability

The data comprising the germplasm SNP genotype database presented in this study are available in Supplementary Table S2. The identity of the SNPs and their chromosomal positions are not publicly available due to protection of the intellectual property right of the ARC to use the KASP SNP panel in the delivery of fingerprinting services. They were named alphabetically from A to Y.

## Supplementary Materials

The following are available online at https://www.mdpi.com/article/10.3390/plants11121546/s1, Figure S1: Complete cluster dendrogram of pairwise genetic distances calculated with the Kosman index of 190 cultivars genotyped with SeqSNP at 500 SNP positions, Figure S2: Complete cluster dendrogram of pairwise genetic distances calculated with the Kosman index of 173 cultivars genotyped with SeqSNP at 25 selected SNP positions, Figure S3: Complete cluster dendrogram of pairwise genetic distances calculated with the Kosman index of 190 cultivars genotyped with SeqSNP at the 21 selected SNP panel positions. Table S1: Potato germplasm list selected for KASP SNP assay verification (78 cultivars), Table S2: Database of SNP genotypes of selected potato cultivars as obtained from SeqSNP and KASP SNP assays at 23 SNP positions.

## Appendix A

**Table A1:** Potato germplasm list selected for SeqSNP genotyping (190 cultivars). GWK, FPD and FL at the “Reason for choice” refers to contributions made by GWK Trading, First Potato Dynamics and Pepsico (FL lines) respectively. Vos gt indicate those overlapping with [11,27]. SA variety list indicate those on the South African potato variety list (Department of Agriculture, Land Reform and Rural Development (DALRRD)).

gDNA	Cultivar Name	Source	Reason for Choice
5	7Four7	Greenhouse—dried	GWK
54	890/20	Field	Did SSR
86	92-0472-042	Field	Did SSR
65	92-466-112	Field	Did SSR
64	94-0530-008 (Freek)	Field	F. Steyn request
100	95-521-126	Field	Did SSR
84	96-0568-002 (Arno)	Field	McCain request
93	96-232-27	Field	Did SSR
95	Abnaki	Field	Choose more
106	Accent	Field	Choose more
192	Adato	In vitro planted in greenhouse	FPD
99	Advira	Field	Choose more
102	Agatha 11	Field	Choose more
19	Agria	Field	Vos gt
30	Alamo	Field	Choose more
94	Alaska 114	Field	Choose more
63	Alcmaria	Field	Vos gt
90	Amalfy	Field	Choose more
35	Amapola	Field	Choose more
101	Amethyst	Field	F. Steyn request
119	Amigo	In vitro	McCain request
120	Anosta	In vitro planted in greenhouse	Vos gt
121	Apache	In vitro	Choose more
60	Arcadia Russet	Field	Choose more
56	Atacama	Field	Choose more
87	Atlantic	Field	Vos gt
61	Atzimba	Field	Choose more
1	Avalanche	Greenhouse	FPD
88	Aviva	Field	SA Variety list
16	Bake King	Field	Choose more
48	Baku	Field	Choose more
122	Barcelona	In vitro	Choose more
6	Belmonda	Greenhouse	GWK
103	Belrus	Field	Choose more
52	Bintje	Field	Vos gt
123	Bordeaux	In vitro	Choose more
51	Boulder	Field	Choose more
124	BP1 (APO)	In vitro	Commercially important
92	BP1 2018	Field	Commercially important
62	Bravo	Field	F. Steyn request
34	Bright	Field	Choose more
125	Bst Galler	In vitro	Choose more
50	Buffelspoort	Field	Commercially important
67	Calibra	Field	SA Variety list
83	Calimero	Field	Choose more
97	Caren	Field	Commercially important
126	Caribou Russet	In vitro	Did SSR
18	Ceasar	Field	Choose more
96	Cedara	Field	Choose more
127	Centennial Russet	In vitro	Choose more
49	Charisma	Field	Choose more
128	Chellah	In vitro	SA Variety list
129	Ciklàmen (Ke.48-5)	In vitro	Choose more
13	Connect	Greenhouse	GWK
130	COO2321	In vitro	Did SSR
32	Corne de Gatte	Field	F. Steyn request
58	Crebella	Field	Did SSR
131	Crispin	In vitro	Did SSR
132	Crop34	In vitro	SA Variety list
133	Crop60 2484	In vitro	McCain request
134	Cwater Russet	In vitro	McCain request
135	Daisy	In vitro	Commercially smaller
55	Dakchip	Field	Choose more
136	Dakota Trailblazer	In vitro	McCain request
137	Darius	In vitro	Commercially important
91	Desiréé	Field	Choose more
66	Devlin	Field	SA Variety list
105	Diamant	Field	Vos gt
20	Diana	Field	Choose more
138	Donata	In vitro	SA Variety list
53 A	Draga (Rascals)	Field	Choose more
116	DTO33	Field	F. Steyn request
139	Earliest of All	In vitro	Choose more
28 (B)	Eldena	Field	Vos gt
140	Elodie	In vitro	Did SSR
44	Elsa	Field	F. Steyn request
22	Ernstoltz	Field	Choose more
31	Eryn	Field	Commercially smaller
141	Esco	In vitro	SA Variety list
42	Escort	Field	Vos gt
111	Esparante	Field	Choose more
142	Estima	In vitro planted in greenhouse	Vos gt
143	Evan	In vitro	SA Variety list
85	Fabian	Field	SA Variety list
25	Fambo	Field	Choose more
46	Fatima	Field	Choose more
43	Felsina	Field	Vos gt
144	Fianna	In vitro planted in greenhouse	Commercially important
145	Figaro	In vitro	SA Variety list
193	FL2006	In vitro planted in greenhouse	FL
194	FL2108	In vitro planted in greenhouse	FL
195	FL2476	In vitro	FL
146	Folva	In vitro	Vos gt
21	Frisia	Field	Vos gt
147	Frodo	In vitro	SA Variety list
148	Gatsby	In vitro	Choose more
39	Gemchip	Field	Choose more
149	Georgina	In vitro	Did SSR
72	Grandifolia	Field	Choose more
150	Hermes	In vitro	Commercially smaller
82	Hertha (Rascals)	Field	Commercially important
69	Hoëvelder	Field	SA Variety list
23	Hudson	Field	Choose more
151	Hydra	In vitro	Choose more
10	IIZA49A1	Greenhouse	GWK
9	IIZASSA5	Greenhouse	GWK
152	Innovator	In vitro planted in greenhouse	Commercially important
153	Irish Gold	In vitro	Did SSR
154	Isle of Jura	In vitro	Choose more
79	Jelly	Field	SA Variety list
115	Jemseg	Field	Choose more
197	Kankan	In vitro planted in greenhouse	Choose more
45	Katahdin	Field	Vos gt
59	Kimb. Choice	Field	Choose more
110	King George	Field	Vos gt
4	King Russet	Greenhouse	GWK
155	Kingsman	In vitro	Did SSR
68	Kingston	Field	Choose more
70	Koos Smit	Field	F. Steyn request
156	La Strada	In vitro	Did SSR
81	Lady Rosetta	Field	Commercially important
11	Lanorma	Greenhouse	GWK
73	Late Harvest	Field	Choose more
74	Lenape	Field	Vos gt
78	Liberator	Field	Choose more
112 (B)	Liseta (Rascals)	Field	Commercially smaller
26	LT 7	Field	F. Steyn request
157	Ludmilla	In vitro	SA Variety list
158	Magnum	In vitro	McCain request
159	Manhattan	In vitro	Did SSR
57	Maradonna	Field	Choose more
109	Marfona	Field	Vos gt
118	Marijke	Field	SA Variety list
75	Maris Piper	Field	Vos gt
107	Marispeer	Field	Choose more
160	Markies	In vitro	FPD
114	Meliose	Field	Choose more
108	Mirakel	Field	Vos gt
117	Mnandi	Field	Commercially smaller
38	Mokgotlong	Field	Choose more
24	Monalisa	Field	Vos gt
76	Mondial	Field	Commercially important
27	Mondial (Rascals)	Field	Commercially important
161	Monica russet	In vitro	McCain request
162	Monte Carlo	In vitro	Vos gt
163	Montreal	In vitro	Choose more
164	Moonlight	In vitro planted in greenhouse	Did SSR
40	Morene	Field	Vos gt
37	Navaan	Field	Choose more
80	Nicola	Field	SA Variety list
113	Nooksack	Field	Choose more
77	Norchip	Field	Choose more
41	Norking Russet	Field	Choose more
7	Noya	Greenhouse	GWK
165	NY 115	In vitro	Choose more
166	Õszirózsa (Ke.31–56)	In vitro	Choose more
167	Ottawa	In vitro	SA Variety list
14	Panamera	Leaf sample—dried	Commercially important
168	PentlandDell	In vitro	Commercially important
12	Prada	Greenhouse	GWK
169	Record	In vitro	Vos gt
170	Renova	In vitro	Choose more
33	Ronn	Field	F. Steyn request
171	Rotharo	In vitro	SA Variety list
3	Royal	Greenhouse	GWK
104	Rua	Field	Choose more
172	Rumba	In vitro	Choose more
173	Russet Burbank	In vitro	SA Variety list
174	Russet Norkotah	In vitro	Vos gt
175	Sabie	In vitro	Choose more
176	Sackfiller	In vitro planted in greenhouse	Choose more
177	Sandvelder	In vitro	SA Variety list
178	Santé	In vitro planted in greenhouse	Choose more
179	Sarpo Mira	In vitro	Vos gt
180	Shepody	In vitro	Commercially smaller
181	Sifra	In vitro	Commercially important
196	Sound	In vitro planted in greenhouse	FPD
182	Spunta	In vitro	SA Variety list
8	Taisiya	Greenhouse—dried	GWK
15	Taurus	Leaf sample—dried	Commercially important
183	Teton Russet	In vitro	McCain request
184	Toronto	In vitro	Choose more
185	Ulster Chief	In vitro planted in greenhouse	Choose more
186	Umatilla Rus	In vitro	Choose more
29	Up to Date 2007	Field	Commercially important
187	Up to Date 2012	In vitro	Commercially important
2	Valor	Greenhouse	Commercially important
189	VanDerPlank	In vitro	Commercially important
190	White Lady	In vitro	Choose more
191	Yukon Gold	In vitro	Vos gt
