# Supplementary material for: Potato Cultivar Identification in South Africa Using a Custom SNP Panel
Source: Plants (Basel). 2022 Jun 10;11(12):1546. doi: 10.3390/plants11121546 (PMC9231109; doi:10.3390/plants11121546)
Supplement: Supplementary file 1 [file plants-11-01546-s001.zip › plants-1713215-supplementary.pdf]

plants

MDPI

Article

Potato Cultivar Identification in South Africa Using a Custom SNP Panel

Inge Gazendam [1,*], Pinkie Mojapelo [1] and Michael W. Bairu [1,2]

Supplementary Materials

Table S1: (pages 1-3). Potato germplasm list selected for KASP SNP assay verification (78 cultivars). GWK, FPD and FL at the "Reason for choice" refer to contributions made by GWK Trading, First Potato Dynamics and Pepsico (FL lines), respectively.

gDNA#	Cultivar Name	Source	Reason for choice
5	7Four7	Greenhouse – dried	GWK
65	92-466-112	Field	Did SSR
64	94-0530-008 (Freek)	Field	F. Steyn request
84	96-0568-002 (Arno)	Field	McCain request
93	96-232-27	Field	Did SSR
192	Adato	*In vitro* planted in greenhouse	FPD
101	Amethyst	Field	F. Steyn request
119	Amigo	*In vitro*	McCain request
1	Avalanche	Greenhouse	FPD
6	Belmonda	Greenhouse	GWK
124	BP1 (APO)	*In vitro*	Commercially important
92	BP1 2018	Field	Commercially important
50	Buffelspoort	Field	Commercially important
97	Caren	Field	Commercially important
13	Connect	Greenhouse	GWK
130	COO2321	*In vitro*	Did SSR
131	Crispin	*In vitro*	Did SSR
132	Crop34	*In vitro*	SA Variety list
133	Crop60 2484	*In vitro*	McCain request
134	Cwater Russet	*In vitro*	McCain request
135	Daisy	*In vitro*	Commercially smaller
136	DakotaTrailblazr	*In vitro*	McCain request
137	Darius	*In vitro*	Commercially important
66	Devlin	Field	SA Variety list
138	Donata	*In vitro*	SA Variety list
116	DTO33	Field	F. Steyn request
140	Elodie	*In vitro*	Did SSR
44	Elsa	Field	F. Steyn request
31	Eryn	Field	Commercially smaller

141	Esco	*In vitro*	SA Variety list
143	Evan	*In vitro*	SA Variety list
85	Fabian	Field	SA Variety list
144	Fianna	*In vitro* planted in greenhouse	Commercially important
193	FL2006	*In vitro* planted in greenhouse	FL
194	FL2108	*In vitro* planted in greenhouse	FL
195	FL2476	*In vitro*	FL
147	Frodo	*In vitro*	SA Variety list
150	Hermes	*In vitro*	Commercially smaller
82	Hertha (Rascals)	Field	Commercially important
10	IIZA49A1	Greenhouse	GWK
9	IIZASSA5	Greenhouse	GWK
152	Innovator	*In vitro* planted in greenhouse	Commercially important
79	Jelly	Field	SA Variety list
4	King Russet	Greenhouse	GWK
155	Kingsman	*In vitro*	Did SSR
70	Koos Smit	Field	F. Steyn request
81	Lady Rosetta	Field	Commercially important
11	Lanorma	Greenhouse	GWK
112	Liseta (Rascals)	Field	Commercially smaller
158	Magnum	*In vitro*	McCain request
118	Marijke	Field	SA Variety list
75	Maris Piper	Field	Vos gt
107	Marispeer	Field	Choose more
160	Markies	*In vitro*	FPD
117	Mnandi	Field	Commercially smaller
76	Mondial	Field	Commercially important
27	Mondial (Rascals)	Field	Commercially important
161	Monica russet	*In vitro*	McCain request
164	Moonlight	*In vitro* planted in greenhouse	Did SSR
7	Noya	Greenhouse	GWK
14	Panamera	Leaf sample - dried	Commercially important
168	PentlandDell	*In vitro*	Commercially important
12	Prada	Greenhouse	GWK
171	Rotharo	*In vitro*	SA Variety list
3	Royal	Greenhouse	GWK
173	Russet Burbank	*In vitro*	SA Variety list
177	Sandvelder	*In vitro*	SA Variety list
180	Shepody	*In vitro*	Commercially smaller
181	Sifra	*In vitro*	Commercially important
196	Sound	*In vitro* planted in greenhouse	FPD
182	Spunta	*In vitro*	SA Variety list

8	Taisiya	Greenhouse - dried	GWK
15	Taurus	Leaf sample - dried	Commercially important
183	Teton Russet	*In vitro*	McCain request
29	Up to Date 2007	Field	Commercially important
187	Up to Date 2012	*In vitro*	Commercially important
2	Valor	Greenhouse	Commercially important
189	VanDerPlank	*In vitro*	Commercially important

Table S2: (pages 1-4). Database of SNP genotypes of selected potato cultivars as obtained from SeqSNP and KASP SNP assays at 23 SNP positions. The reference allele dosage is indicated as a number between 0 and 4.

			KASP SNP assay																						
gDNA#	Cultivar name	Genotype	A	C	D	E	F	G	H	J	K	L	M	N	O	P	Q	R	S	T	U	V	W	X	Y
5	7Four7	SeqSNP	4	0	3	3	3	0	3	1	3	3	2	2	3	1	3	2	3	2	0	3	2	2	3
5	7Four7	KASP	4	0	3	3	3	0	3	1	3	3	2	2	3	1	3	2	3	2	0	3	2	2	3
54	890/20	SeqSNP	3	1	3	1	2	1	3	3	4	2	3	4	2	1	1	2	2	2	2	2	3	3	1
86	92-0472-042	SeqSNP	1	1	1	1	2	2	3	1	1	2	2	3	2	1	1	2	1	2	0	1	2	2	3
65	92-466-112	SeqSNP	3	2	1	0	4	1	0	1	1	0	3	2	3	1	0	2	3	2	2	1	3	3	3
65	92-466-112	KASP	3	2	1	0	4	1	0	1	1	0	3	2	3	1	0	2	3	2	2	1	3	3	3
64	94-0530-008 (Freek)	SeqSNP	2	0	2	1	2	2	2	1	2	2	3	2	4	2	1	1	1	1	1	1	2	3	3
64	94-0530-008 (Freek)	KASP	2	0	2	1	2	2	2	1	2	2	3	2	4	2	1	1	1	1	1	1	2	3	3
100	95-521-126	SeqSNP	2	0	1	0	2	3	2	1	1	2	1	2	3	0	1	3	3	3	2	0	3	2	3
84	96-0568-002 (Arno)	SeqSNP	3	4	2	2	1	2	3	2	1	4	0	3	1	1	3	2	1	4	3	1	2	2	4
84	96-0568-002 (Arno)	KASP	3	4	2	2	1	2	3	2	1	4	0	3	1	1	3	2	1	4	3	1	2	2	4
93	96-232-27	SeqSNP	3	3	0	0	1	1	1	2	1	1	1	1	2	2	1	2	2	1	2	1	1	3	3
93	96-232-27	KASP	3	3	0	0	1	1	1	2	1	1	1	1	2	2	1	2	2	1	2	1	1	3	3
95	Abnaki	SeqSNP	1	1	0	1	2	0	0	2	2	0	2	1	2	2	3	0	3	2	2	2	2	2	4
106	Accent	SeqSNP	2	0	2	2	2	2	3	3	4	1	2	2	2	2	2	4	2	2	2	3	1	2	2
192	Adato	SeqSNP	3	3	2	3	4	4	3	0	1	3	1	1	1	1	1	1	4	1	1	3	1	4	3
192	Adato	KASP	3	3	2	3	4	4	3	0	1	3	1	1	1	1	1	1	4	1	1	3	1	4	3
99	Advira	SeqSNP	3	1	3	1	2	1	3	3	4	2	3	4	2	1	1	2	2	2	2	2	3	3	1
102	Agatha 11	SeqSNP	1	2	0	4	2	1	3	2	3	1	0	2	4	1	2	0	4	1	1	1	4	1	1
19	Agria	SeqSNP	2	1	2	2	3	4	3	3	2	2	3	1	1	2	3	2	2	3	1	2	3	3	2
30	Alamo	SeqSNP	3	2	1	4	3	0	1	3	3	2	2	2	1	1	1	1	3	1	3	2	2	1	3
94	Alaska 114	SeqSNP	1	2	0	2	4	1	3	4	2	0	3	1	2	2	2	0	3	1	3	1	1	2	2
63	Alcmaria	SeqSNP	3	2	1	2	3	2	3	2	2	2	4	3	1	1	1	1	1	2	3	2	3	2	2
90	Amalfy	SeqSNP	1	3	1	1	2	1	3	2	2	1	1	3	3	1	2	2	2	1	1	3	1	1	2
35	Amapola	SeqSNP	3	4	0	2	3	3	2	3	3	2	1	2	1	2	0	0	3	3	1	2	2	2	1
101	Amethyst	SeqSNP	4	2	1	2	3	2	1	1	2	2	1	2	2	0	2	1	1	1	0	3	3	3	2
101	Amethyst	KASP	4	2	1	2	3	2	1	1	2	2	1	2	2	0	2	1	1	1	0	3	3	3	2
119	Amigo	SeqSNP	3	1	0	3	3	4	0	2	2	3	3	1	3	2	2	3	1	2	2	3	3	3	2
119	Amigo	KASP	3	1	0	3	3	4	0	2	2	2	3	1	3	2	2	3	1	2	2	3	3	3	2
120	Anosta	SeqSNP	1	3	3	1	1	1	3	2	1	3	1	2	2	1	4	2	1	1	2	3	3	2	1
121	Apache	SeqSNP	1	2	3	4	2	2	3	2	2	2	3	3	3	1	1	3	4	3	0	1	4	2	3
60	Arcadia Russet	SeqSNP	2	3	1	3	2	0	2	1	0	3	3	2	3	3	4	2	2	2	2	2	1	1	3
56	Atacama	SeqSNP	2	2	3	2	2	3	4	2	1	1	2	4	4	2	1	3	0	4	1	1	2	3	3
56	Atacama	KASP	NA	NA	NA	NA	NA	1	2	NA	3	2	NA	2	NA	NA	NA	NA	3	2	NA	NA	NA	NA	NA
87	Atlantic	SeqSNP	2	0	0	0	0	4	3	2	2	2	2	2	2	3	1	1	1	2	1	1	3	1	2
87	Atlantic	KASP	NA	0	0	0	0	4	NA	NA	NA	NA	NA	NA	NA	3	NA	NA	1	NA	NA	NA	NA	1	NA
61	Atzimba	SeqSNP	0	3	2	1	3	2	1	3	3	3	1	1	3	1	2	2	2	2	2	2	3	1	4
1	Avalanche	SeqSNP	2	3	3	2	3	2	3	2	3	0	2	3	1	0	2	0	1	1	1	2	2	2	2
1	Avalanche	KASP	2	3	3	2	3	2	3	2	3	0	2	3	1	0	2	0	1	1	1	2	2	2	2
88	Aviva	SeqSNP	1	3	3	1	2	0	3	2	1	1	0	3	3	2	2	2	2	2	3	0	2	4	2
16	Bake King	SeqSNP	2	2	0	0	1	2	3	3	2	3	3	3	4	3	3	3	1	2	1	1	2	3	3
48	Baku	SeqSNP	2	1	3	3	3	1	4	3	3	2	2	1	4	2	3	3	2	1	1	1	0	2	4
122	Barcelona	SeqSNP	2	1	3	2	1	2	4	2	2	3	2	2	1	1	0	2	1	1	3	4	2	3	2
6	Belmonda	SeqSNP	1	3	3	4	2	3	1	3	4	3	3	2	1	2	2	1	2	1	1	2	2	2	3
6	Belmonda	KASP	1	3	3	4	2	3	1	3	4	3	3	2	1	2	1	1	2	1	1	2	2	2	3
103	Belrus	SeqSNP	3	3	3	2	3	1	3	1	0	3	3	1	2	2	1	1	3	3	3	1	0	2	4
52	Bintje	SeqSNP	4	3	3	3	2	1	3	1	3	2	3	0	3	2	2	2	3	2	2	2	2	4	3
123	Bordeaux	SeqSNP	2	1	2	1	3	0	2	3	1	1	3	0	3	4	1	1	2	2	2	1	1	3	3
51	Boulder	SeqSNP	1	0	0	2	1	2	2	1	2	1	1	1	1	1	3	2	1	0	1	1	3	2	1
124	BP1 (APO)	SeqSNP	2	3	1	2	1	2	3	1	3	2	1	1	4	2	3	1	2	1	0	0	2	2	4
124	BP1 (APO)	KASP	2	3	1	2	1	2	2	1	3	2	1	1	4	2	3	1	2	1	0	0	2	2	4
124_d	BP1 (APO)_d	SeqSNP	2	3	1	2	1	2	3	1	3	2	1	1	4	2	3	1	2	1	0	0	2	2	4
124_d	BP1 (APO)_d	KASP	2	3	1	2	1	2	2	1	3	2	1	1	4	2	3	1	2	1	0	0	2	2	4
92	BP1 2018	SeqSNP	2	3	1	2	1	2	2	1	3	2	1	1	4	2	3	1	2	1	0	0	2	2	4
92	BP1 2018	KASP	2	3	1	2	1	2	2	1	3	2	1	1	4	2	3	1	2	1	0	0	2	2	4
92_d	BP1 2018_d	SeqSNP	2	3	1	2	1	2	2	1	3	2	1	1	4	2	3	1	2	1	0	0	2	2	4
92_d	BP1 2018_d	KASP	2	3	1	2	1	2	2	1	3	2	1	1	4	2	3	1	2	1	0	0	2	2	4
62	Bravo	SeqSNP	3	2	1	2	1	1	3	0	1	2	2	1	3	2	3	2	2	2	0	1	1	1	3
34	Bright	SeqSNP	2	2	3	1	1	2	3	1	3	2	2	3	3	1	2	0	4	2	1	3	2	2	3
125	Bst Galler	SeqSNP	2	1	2	2	1	0	3	1	0	2	2	1	3	3	3	4	1	1	3	2	1	2	2
50	Buffelspoort	SeqSNP	1	2	0	3	3	3	2	1	1	2	1	3	0	0	3	1	3	2	3	1	4	0	2
50	Buffelspoort	KASP	1	2	0	3	3	3	2	1	1	2	1	3	0	0	2	1	3	2	3	1	4	0	2
67	Calibra	SeqSNP	3	2	2	2	1	2	2	0	0	2	1	2	4	0	2	1	3	1	2	1	2	3	3
83	Calimero	SeqSNP	2	3	1	2	1	2	3	1	3	2	1	1	4	2	3	1	2	1	0	0	2	2	4
97	Caren	SeqSNP	2	2	1	2	1	2	3	2	3	3	1	1	2	0	3	1	3	2	2	2	2	1	4
97	Caren	KASP	2	2	1	2	1	2	3	NA	3	3	1	1	2	0	3	1	3	2	2	2	2	1	4
126	Caribou Russet	SeqSNP	2	2	3	2	1	1	3	4	2	1	1	2	2	2	1	1	3	0	0	1	3	1	0
126	Caribou Russet	KASP	2	NA	NA	NA	1	NA	NA	4	2	1	1	NA	NA	NA	1	NA	NA	0	0	1	3	1	0
18	Ceasar	SeqSNP	2	1	2	3	3	4	2	2	3	2	3	1	3	3	3	1	1	2	2	2	2	4	1
96	Cedara	SeqSNP	3	2	0	2	1	1	0	2	2	2	2	1	2	1	0	2	2	3	2	2	2	2	2
127	Centennial Russet	SeqSNP	2	1	2	3	4	1	2	4	2	3	1	1	1	1	2	0	4	0	2	3	3	2	4
127	Centennial Russet	KASP	NA	1	NA	NA	4	NA	2	4	NA	NA	NA	NA	NA	NA	NA	NA	4	0	NA	NA	NA	NA	NA
49	Charisma	SeqSNP	2	3	1	2	3	1	2	3	2	0	2	1	1	2	0	1	2	2	4	3	2	3	2

gDNA#	Cultivar name	Genotype	A	C	D	E	F	G	H	J	K	L	M	N	O	P	Q	R	S	T	U	V	W	X	Y
			KASP SNP assay																						
128	Chellah	SeqSNP	2	4	2	2	3	1	3	2	3	2	3	3	3	0	2	2	3	2	3	1	3	3	3
129	Ciklàmen(Ke.48-5)	SeqSNP	1	2	1	2	2	1	3	2	3	3	0	3	3	2	2	2	4	2	2	2	1	3	1
13	Connect	SeqSNP	0	1	3	2	1	1	2	0	3	2	2	3	0	1	1	2	3	2	2	1	3	3	1
13	Connect	KASP	0	1	2	2	1	1	1	0	3	2	3	3	1	1	1	2	3	2	2	1	3	3	1
130	COO2321	SeqSNP	2	1	1	1	3	0	1	1	3	2	2	1	2	3	4	1	3	2	2	2	2	2	2
130	COO2321	KASP	2	1	1	1	3	0	1	1	3	2	2	1	2	3	4	1	3	2	2	2	2	2	2
32	Corne de Gatte	SeqSNP	2	2	3	2	3	1	3	1	0	3	2	3	2	2	2	2	2	3	2	2	4	1	3
58	Crebella	SeqSNP	3	3	1	3	4	1	1	1	4	2	3	3	1	1	2	3	2	1	3	3	0	3	2
131	Crispin	SeqSNP	3	0	2	2	2	1	1	3	4	1	3	0	2	2	2	1	1	2	3	3	2	2	1
131	Crispin	KASP	3	0	2	2	2	1	1	3	4	1	3	0	2	2	2	1	1	2	3	3	2	2	1
132	Crop34	SeqSNP	1	2	2	3	0	2	2	2	2	2	1	2	2	2	3	1	2	2	3	1	2	3	2
132	Crop34	KASP	1	2	2	3	0	2	2	2	2	2	1	2	2	2	3	1	2	2	3	1	2	3	2
133	Crop60 2484	SeqSNP	2	2	3	3	3	0	3	1	2	2	1	2	2	1	2	1	3	2	0	1	2	1	1
133	Crop60 2484	KASP	2	2	3	3	3	0	3	1	2	2	1	2	2	1	2	1	3	2	0	1	2	1	1
134	Cwater Russet	SeqSNP	1	1	1	2	3	1	3	3	3	1	1	2	2	3	3	1	4	1	1	2	1	4	2
134	Cwater Russet	KASP	1	1	1	2	3	1	2	3	3	1	1	2	2	3	3	1	4	1	1	2	1	4	2
135	Daisy	SeqSNP	2	3	1	1	2	3	3	1	0	3	0	3	2	2	0	3	3	1	2	2	2	3	2
135	Daisy	KASP	2	NA	1	1	2	3	3	NA	0	3	0	3	2	2	0	3	3	1	2	2	2	3	2
55	Dakchip	SeqSNP	2	3	1	2	1	2	3	1	3	2	1	0	4	3	3	1	2	1	0	0	2	2	4
136	DakotaTrailblazr	SeqSNP	2	0	1	2	2	2	3	2	3	1	2	0	1	2	2	2	1	2	3	0	2	1	2
136	DakotaTrailblazr	KASP	2	0	1	NA	2	2	2	2	3	1	2	0	1	2	2	2	1	2	3	0	2	1	2
137	Darius	SeqSNP	3	2	1	3	0	0	1	3	3	1	0	2	2	3	2	2	4	2	2	2	3	2	4
137	Darius	KASP	2	2	1	3	0	0	1	3	3	1	0	2	2	3	2	2	4	2	2	2	3	2	4
91	Desiréé	SeqSNP	0	3	2	2	2	3	1	3	1	2	1	3	3	2	2	3	2	2	1	3	1	2	2
66	Devlin	SeqSNP	2	3	2	3	1	1	1	3	2	2	0	2	2	1	0	2	3	2	2	2	0	2	3
66	Devlin	KASP	2	3	2	3	1	1	1	3	2	2	0	2	2	1	0	2	3	2	2	2	0	2	3
105	Diamant	SeqSNP	2	3	1	4	3	1	3	1	3	1	3	3	3	2	3	2	4	2	3	3	0	2	3
20	Diana	SeqSNP	0	2	1	1	2	3	1	1	2	2	2	4	2	2	3	2	3	2	1	3	1	3	3
138	Donata	SeqSNP	1	2	2	2	2	1	2	3	2	4	3	2	2	2	1	1	1	2	0	1	4	2	2
138	Donata	KASP	1	2	2	2	2	1	1	3	2	4	4	2	2	2	1	1	1	2	0	1	4	2	2
53	Draga (Rascals)	SeqSNP	2	0	3	4	3	0	0	2	2	3	1	2	0	1	3	1	3	2	2	2	4	3	1
53	Draga (Rascals)	KASP	NA	0	NA	4	NA	NA	0	2	NA	NA	1	NA	0	NA	NA	NA	NA	NA	NA	NA	4	NA	1
116	DTO33	SeqSNP	2	1	1	2	3	0	2	3	3	4	4	4	3	3	3	3	3	3	1	1	1	3	2
116	DTO33	KASP	2	1	1	2	3	0	1	3	3	4	4	4	3	3	3	3	3	3	1	1	1	3	2
139	Earliest of All	SeqSNP	1	3	1	3	3	1	1	2	4	2	0	2	3	2	2	0	2	1	2	1	1	2	3
28	Eldena	SeqSNP	0	3	1	3	2	1	2	1	2	1	3	0	3	2	2	2	3	2	1	2	4	2	2
140	Elodie	SeqSNP	4	1	2	2	2	2	4	2	3	3	2	2	1	1	2	3	3	1	0	3	2	2	2
140	Elodie	KASP	4	1	2	2	2	2	4	2	3	3	3	2	1	1	2	3	3	1	0	3	2	2	2
44	Elsa	SeqSNP	0	4	2	1	2	1	0	3	2	3	4	2	1	3	1	1	2	2	1	3	1	2	3
44	Elsa	KASP	0	4	2	1	2	1	0	3	2	3	4	2	1	3	1	1	3	2	1	3	1	2	3
22	Ernstoltz	SeqSNP	1	2	2	2	2	2	0	1	4	1	2	3	1	4	3	2	3	1	4	1	3	2	2
31	Eryn	SeqSNP	2	3	1	4	0	1	3	3	1	1	2	1	3	3	1	2	4	3	2	2	2	3	2
31	Eryn	KASP	2	NA	1	4	0	1	2	3	1	1	3	1	3	3	1	2	4	3	2	2	2	3	2
141	Esco	SeqSNP	2	3	1	1	2	3	2	3	2	2	3	0	2	1	1	1	2	2	2	3	2	1	2
141	Esco	KASP	2	3	1	1	2	3	1	3	2	2	3	0	2	1	1	1	2	2	2	3	2	1	2
42	Escort	SeqSNP	2	3	1	1	2	3	2	3	2	2	3	0	2	1	1	1	2	2	2	3	2	1	2
111	Esparante	SeqSNP	2	1	3	3	2	1	2	1	3	2	2	2	1	3	2	2	2	3	2	4	2	4	3
142	Estima	SeqSNP	4	2	3	2	3	2	2	2	1	2	3	2	2	2	2	2	2	1	1	1	2	3	4
143	Evan	SeqSNP	2	3	2	2	1	1	2	1	0	1	2	1	1	2	3	2	4	3	2	3	2	0	2
143	Evan	KASP	2	3	2	2	1	1	1	1	0	1	2	1	1	2	3	2	4	3	2	3	2	0	2
85	Fabian	SeqSNP	3	2	0	2	3	1	1	2	3	2	2	2	4	2	2	3	3	1	0	0	1	3	2
85	Fabian	KASP	3	2	0	2	3	1	1	2	3	2	2	2	4	2	2	2	3	1	0	0	1	3	2
25	Fambo	SeqSNP	2	2	3	3	2	1	1	2	0	1	1	2	3	2	1	2	1	2	1	2	2	4	4
46	Fatima	SeqSNP	2	2	1	2	3	3	1	2	2	3	1	1	3	3	2	0	1	2	1	2	3	4	4
43	Felsina	SeqSNP	0	2	4	3	1	1	2	2	4	2	1	3	2	1	1	2	1	1	3	4	3	2	1
43	Felsina	KASP	0	NA	4	NA	1	NA	NA	NA	NA	NA	NA	NA	NA	NA	NA	NA	1	NA	3	4	NA	NA	1
144	Fianna	SeqSNP	1	2	2	3	1	1	1	2	2	0	2	1	2	2	2	1	2	2	2	3	3	4	2
144	Fianna	KASP	1	2	2	3	1	0	1	2	2	0	2	1	2	2	2	1	2	2	2	3	3	4	2
145	Figaro	SeqSNP	1	3	2	1	1	1	3	1	4	2	3	2	1	2	1	2	1	1	2	2	3	3	2
193	FL2006	SeqSNP	2	2	1	1	0	4	1	3	3	2	2	1	1	2	1	0	2	1	2	3	3	1	1
193	FL2006	KASP	2	2	1	1	0	4	1	3	3	2	2	1	1	2	1	0	2	1	2	3	3	1	1
194	FL2108	SeqSNP	2	2	2	2	2	3	0	0	2	2	1	4	2	0	2	1	3	1	1	2	3	3	2
194	FL2108	KASP	2	2	2	2	2	3	0	0	2	2	2	4	2	0	2	1	3	1	1	2	3	3	2
195	FL2476	SeqSNP	3	0	2	2	3	4	1	2	1	2	2	3	4	1	2	3	4	3	0	2	4	4	3
195	FL2476	KASP	3	0	2	2	3	4	1	2	1	2	3	3	4	1	2	3	4	3	0	2	4	4	3
146	Folva	SeqSNP	1	2	3	1	1	2	2	0	3	2	1	1	0	0	0	3	2	1	2	2	3	2	3
21	Frisia	SeqSNP	2	2	1	2	3	3	1	2	2	3	1	1	3	3	2	0	1	2	1	2	3	4	4
147	Frodo	SeqSNP	1	1	2	3	2	0	3	2	3	3	2	0	2	3	2	3	4	1	2	0	3	2	2
147	Frodo	KASP	1	1	2	3	2	0	2	2	3	3	2	0	2	3	2	3	4	1	2	0	3	2	2
148	Gatsby	SeqSNP	2	1	1	1	2	2	3	2	2	2	2	2	1	0	1	2	1	2	2	3	2	3	4
39	Gemchip	SeqSNP	1	0	1	1	3	1	3	2	4	2	2	2	3	2	3	2	3	1	3	2	3	2	2
149	Georgina	SeqSNP	3	2	3	2	3	2	3	0	4	3	3	3	2	3	3	3	1	1	1	2	2	2	2
72	Grandifolia	SeqSNP	1	2	2	3	3	3	1	1	3	1	3	1	2	2	1	0	3	1	2	2	4	1	3

			KASP SNP assay																						
gDNA#	Cultivar name	Genotype	A	C	D	E	F	G	H	J	K	L	M	N	O	P	Q	R	S	T	U	V	W	X	Y
150	Hermes	SeqSNP	3	2	4	2	2	2	0	2	3	1	2	2	4	1	3	2	3	3	1	1	2	3	1
150	Hermes	KASP	3	2	4	2	2	2	0	2	3	1	4	2	4	1	3	2	3	3	1	1	2	3	1
82	Hertha (Rascals)	SeqSNP	1	1	2	2	2	2	3	2	3	4	3	3	3	2	1	2	2	2	1	2	4	2	4
82	Hertha (Rascals)	KASP	1	1	2	2	2	2	2	2	3	4	3	3	3	2	1	2	2	2	1	2	4	2	4
69	Hoëvelder	SeqSNP	1	2	1	1	1	2	0	2	1	1	1	2	2	3	1	2	4	2	1	1	1	2	3
23	Hudson	SeqSNP	3	1	2	1	3	1	2	2	2	0	0	1	3	2	3	2	3	3	0	3	1	3	3
151	Hydra	SeqSNP	2	2	1	0	4	2	3	3	3	2	1	2	2	0	1	2	2	0	2	2	2	1	1
10	IIZA49A1	SeqSNP	2	2	3	1	4	1	2	2	2	1	1	2	1	2	3	1	2	2	1	2	2	1	3
10	IIZA49A1	KASP	2	2	3	1	4	1	2	2	2	1	1	2	1	2	3	1	2	2	1	2	2	1	3
9	IIZASSA5	SeqSNP	2	3	2	0	3	2	3	2	4	1	3	1	2	1	2	1	2	2	1	2	1	3	3
9	IIZASSA5	KASP	2	3	2	0	3	2	3	2	4	1	3	1	2	1	2	1	2	2	1	2	1	3	3
152	Innovator	SeqSNP	1	4	1	1	1	2	3	0	2	3	4	2	0	1	3	3	2	2	1	2	1	1	3
152	Innovator	KASP	1	4	1	1	1	2	2	0	2	3	4	2	1	1	3	3	2	2	1	2	1	1	3
153	Irish Gold	SeqSNP	3	2	4	3	4	3	3	2	1	2	3	2	3	3	3	1	1	2	2	2	2	2	3
154	Isle of Jura	SeqSNP	2	2	3	1	2	2	3	2	3	2	3	2	1	0	1	2	0	0	2	0	3	0	2
79	Jelly	SeqSNP	4	2	1	0	1	3	2	1	3	2	2	2	0	2	1	0	2	2	3	3	1	1	2
79	Jelly	KASP	4	2	1	0	1	3	2	1	3	2	3	2	0	2	1	0	2	2	3	3	1	1	2
115	Jemseg	SeqSNP	1	2	2	3	4	2	1	3	3	2	3	1	3	3	2	2	0	2	0	1	3	0	3
197	Kankan	SeqSNP	2	2	0	3	3	0	2	3	2	3	0	3	3	2	3	2	3	0	1	1	1	3	1
45	Katahdin	SeqSNP	2	2	1	1	3	1	3	3	3	2	2	2	4	1	2	0	2	1	1	0	2	2	2
59	Kimb. Choice	SeqSNP	3	4	2	2	2	0	2	3	1	1	1	3	3	3	1	3	4	2	2	1	2	3	3
110	King George	SeqSNP	2	4	2	2	1	1	3	1	4	2	3	1	4	2	3	3	2	2	0	1	3	3	2
110	King George	KASP	NA	4	NA	NA	1	NA	NA	1	4	NA	4	1	4	NA	NA	3	NA	NA	NA	NA	NA	NA	NA
4	King Russet	SeqSNP	4	4	1	1	0	1	1	0	1	2	4	0	0	2	2	3	2	2	2	3	3	2	1
4	King Russet	KASP	4	4	1	1	0	1	1	0	1	2	4	0	0	2	2	3	2	2	2	3	3	2	1
155	Kingsman	SeqSNP	1	0	3	3	3	4	2	4	3	2	2	1	2	1	2	0	1	2	2	2	2	3	2
155	Kingsman	KASP	1	0	3	3	3	4	2	4	3	2	2	1	2	1	2	0	1	2	2	2	2	3	2
68	Kingston	SeqSNP	1	2	1	2	1	2	1	1	3	1	2	2	2	0	1	2	2	1	0	1	3	1	1
70	Koos Smit	SeqSNP	2	2	3	2	2	1	4	2	4	1	2	3	3	2	2	2	3	3	4	3	1	3	0
70	Koos Smit	KASP	2	3	3	2	3	1	3	0	3	2	1	3	2	2	3	2	2	2	2	2	2	3	3
156	La Strada	SeqSNP	3	0	3	2	4	1	0	1	3	1	1	1	1	1	0	3	2	0	2	2	2	2	3
81	Lady Rosetta	SeqSNP	2	2	3	2	2	1	4	2	4	1	2	3	3	2	2	2	3	3	4	3	1	3	0
81	Lady Rosetta	KASP	2	2	3	2	2	1	4	2	4	1	2	3	3	2	2	2	3	3	4	3	1	3	0
11	Lanorma	SeqSNP	1	1	3	1	2	2	3	1	2	1	2	2	2	4	3	1	2	3	3	2	1	4	3
11	Lanorma	KASP	1	1	3	1	2	2	3	1	2	1	2	2	2	4	3	1	2	3	3	2	1	4	3
73	Late Harvest	SeqSNP	2	2	2	2	3	2	2	1	3	4	2	2	2	1	2	3	1	1	2	2	1	3	2
74	Lenape	SeqSNP	2	2	2	2	3	2	2	1	3	4	2	2	2	1	2	3	1	1	2	2	1	3	2
78	Liberator	SeqSNP	3	2	2	2	0	2	2	1	3	1	2	1	0	4	2	2	2	1	2	3	3	2	1
112	Liseta (Rascals)	SeqSNP	3	2	2	2	4	2	4	2	0	2	1	1	3	2	2	1	2	1	1	3	2	3	2
112	Liseta (Rascals)	KASP	3	2	1	2	4	2	4	2	0	2	1	1	3	2	2	1	2	1	1	3	2	3	2
26	LT 7	SeqSNP	3	1	1	1	1	3	3	3	1	3	0	3	1	1	3	3	2	2	3	0	2	3	3
157	Ludmilla	SeqSNP	2	2	2	1	1	1	1	0	2	2	4	1	0	1	2	2	1	1	0	3	2	2	2
158	Magnum	SeqSNP	2	1	1	3	2	3	3	1	3	3	2	2	0	2	3	3	2	4	1	2	2	4	4
158	Magnum	KASP	2	NA	1	3	2	3	2	1	3	3	4	2	0	2	3	3	2	4	1	2	2	4	4
159	Manhattan	SeqSNP	2	1	2	2	3	3	2	1	1	3	1	2	1	1	4	2	1	2	1	0	3	4	4
159	Manhattan	KASP	NA	1	NA	NA	NA	3	2	NA	1	NA	2	NA	1	1	4	NA	1	NA	1	0	3	4	4
57	Maradonna	SeqSNP	3	0	1	3	3	1	3	1	3	2	4	2	2	1	4	3	3	2	3	3	3	2	2
109	Marfona	SeqSNP	2	3	2	3	2	2	2	3	4	1	3	1	1	3	3	2	3	2	1	3	1	2	3
118	Marijke	SeqSNP	1	3	2	0	2	1	3	1	2	2	1	1	3	2	2	2	3	1	2	4	1	4	2
118	Marijke	KASP	1	3	2	0	2	1	2	1	2	2	2	1	3	2	2	2	3	1	2	4	1	4	2
75	Maris Piper	SeqSNP	4	3	3	3	2	0	3	1	3	3	2	3	4	2	2	2	3	3	1	1	2	1	2
75	Maris Piper	KASP	4	3	3	3	2	0	2	1	3	3	3	3	4	2	2	2	NA	3	1	1	2	1	2
107	Marispeer	SeqSNP	4	3	3	3	2	0	3	1	3	3	2	3	4	2	2	2	3	3	1	1	2	1	2
107	Marispeer	KASP	4	3	3	3	2	0	2	1	3	3	2	3	4	2	2	2	3	3	1	1	2	1	2
160	Markies	SeqSNP	1	3	2	2	2	3	1	3	3	2	2	0	2	2	1	3	1	2	2	3	3	4	1
160	Markies	KASP	1	3	1	2	2	3	1	3	3	2	3	0	2	2	1	3	1	2	2	3	3	4	1
114	Meliose	SeqSNP	2	2	3	3	2	2	2	2	1	3	2	2	4	2	4	2	2	1	2	3	3	3	4
108	Mirakel	SeqSNP	2	1	0	2	2	1	2	2	4	2	2	3	3	2	3	2	2	2	3	4	0	2	3
117	Mnandi	SeqSNP	1	3	2	3	2	1	3	0	3	2	2	2	1	2	0	1	2	2	3	0	2	3	3
117	Mnandi	KASP	1	3	2	3	2	1	2	1	3	2	2	2	1	2	0	1	3	2	3	0	2	3	3
38	Mokgotlong	SeqSNP	2	2	1	0	4	2	3	3	3	2	1	2	2	0	1	2	2	0	2	2	2	1	2
24	Monalisa	SeqSNP	1	2	2	3	3	3	1	2	2	2	4	2	3	2	4	1	0	0	2	2	0	4	2
24	Monalisa	KASP	NA	NA	NA	NA	NA	3	NA	NA	NA	NA	4	NA	NA	NA	4	NA	0	0	NA	NA	0	4	NA
76	Mondial	SeqSNP	2	2	2	1	3	1	4	1	0	3	3	1	2	3	2	1	1	2	2	3	1	3	3
76	Mondial	KASP	2	2	2	2	3	1	4	1	0	3	3	1	2	3	2	1	1	2	2	3	1	3	3
27	Mondial (Rascals)	SeqSNP	2	2	2	1	3	1	4	1	0	3	3	1	2	3	2	1	1	2	2	3	1	3	3
27	Mondial (Rascals)	KASP	2	2	2	1	3	1	4	1	0	3	3	1	2	3	2	1	1	2	2	3	1	3	3
27_d	Mondial (Rascals)_d	SeqSNP	2	2	2	1	3	1	4	1	0	3	3	1	2	3	2	1	1	2	2	3	1	3	3
27_d	Mondial (Rascals)_d	KASP	2	2	2	1	3	1	4	1	0	3	3	1	2	3	2	1	1	2	2	3	1	3	3
76_d	Mondial_d	SeqSNP	2	2	2	1	3	1	4	1	0	3	3	1	2	3	2	1	1	2	2	3	1	3	3
76_d	Mondial_d	KASP	2	2	2	1	3	1	4	1	0	3	3	1	2	3	2	1	1	2	2	3	1	3	3
161	Monica russet	SeqSNP	1	4	1	1	1	2	3	0	2	3	4	2	1	1	3	3	2	2	1	2	1	1	3
161	Monica russet	KASP	1	4	1	1	1	2	2	0	2	3	4	2	1	1	3	3	2	2	1	2	1	1	3

			KASP SNP assay																						
**gDNA#**	**Cultivar name**	**Genotype**	A	C	D	E	F	G	H	J	K	L	M	N	O	P	Q	R	S	T	U	V	W	X	Y
162	Monte Carlo	SeqSNP	3	2	3	3	0	0	3	1	3	0	2	1	2	0	2	4	3	3	3	3	2	3	2
163	Montreal	SeqSNP	0	2	3	2	1	3	1	2	2	2	2	2	3	3	3	4	3	2	2	2	1	2	2
164	Moonlight	SeqSNP	1	2	4	0	4	0	3	1	2	3	3	2	3	1	1	2	4	3	2	1	1	3	4
164	Moonlight	KASP	1	2	4	0	4	0	3	1	2	3	3	2	3	1	1	2	4	3	2	1	1	3	4
40	Morene	SeqSNP	2	2	4	4	2	0	3	3	2	2	1	2	2	2	2	3	2	1	4	2	3	2	2
37	Navaan	SeqSNP	0	3	4	2	2	1	3	2	2	1	1	2	2	1	0	2	2	2	1	0	2	1	2
80	Nicola	SeqSNP	1	2	3	3	2	2	3	0	2	3	3	2	1	1	3	2	3	2	2	4	3	3	1
113	Nooksack	SeqSNP	1	2	3	3	2	2	3	0	2	3	3	2	1	1	3	2	3	2	2	4	3	3	1
77	Norchip	SeqSNP	3	2	2	2	4	2	4	2	0	2	1	1	3	2	2	1	2	1	1	3	2	3	2
41	Norking Russet	SeqSNP	2	3	3	2	3	2	3	0	3	3	1	3	2	2	3	2	2	2	2	2	2	3	4
7	Noya	SeqSNP	2	2	2	3	4	2	2	2	1	3	4	2	1	2	3	2	3	3	3	1	2	3	3
7	Noya	KASP	2	2	2	3	4	2	2	2	1	3	4	2	1	2	3	2	3	3	3	1	2	3	3
165	NY 115	SeqSNP	2	1	1	2	2	2	2	2	3	2	2	0	0	4	3	2	3	2	2	2	3	3	2
166	Õszirózsa(Ke.31-56)	SeqSNP	1	3	3	3	2	2	2	2	3	3	3	2	3	3	2	4	2	0	3	1	1	4	2
167	Ottawa	SeqSNP	2	3	2	1	3	3	2	1	3	3	0	1	3	2	1	2	1	2	3	2	3	4	3
14	Panamera	SeqSNP	3	0	1	1	3	3	3	3	4	2	1	2	3	1	3	0	3	3	1	4	1	3	3
14	Panamera	KASP	3	0	2	1	3	3	2	3	4	2	1	2	3	1	3	0	3	3	1	4	1	3	3
168	PentlandDell	SeqSNP	2	1	3	2	3	0	3	1	1	3	0	1	2	0	3	3	2	2	0	2	1	4	3
168	PentlandDell	KASP	2	1	3	2	3	0	3	1	1	3	0	1	2	0	3	3	2	2	0	2	1	4	3
12	Prada	SeqSNP	3	2	1	1	2	3	2	2	2	1	3	1	1	1	2	1	2	2	3	3	2	4	0
12	Prada	KASP	3	NA	1	1	2	3	1	2	2	1	3	1	1	1	2	1	2	2	3	3	2	4	0
169	Record	SeqSNP	2	3	2	4	2	2	1	3	2	2	2	1	4	3	1	3	3	2	1	3	2	1	2
170	Renova	SeqSNP	3	2	2	3	4	1	1	2	1	3	1	3	1	2	2	1	1	2	3	2	2	2	2
33	Ronn	SeqSNP	1	1	3	0	4	2	1	1	1	2	3	2	4	1	0	2	2	3	1	0	2	3	3
171	Rotharo	SeqSNP	0	1	2	1	2	2	1	1	2	2	0	2	4	2	3	0	3	1	0	0	2	2	3
171	Rotharo	KASP	0	1	2	1	2	2	1	1	2	2	0	2	4	2	3	0	3	1	0	0	2	2	3
3	Royal	SeqSNP	3	4	2	1	1	3	3	2	2	2	3	1	1	2	1	2	2	3	1	3	4	1	3
3	Royal	KASP	3	4	2	1	1	3	3	2	2	2	3	1	1	2	1	2	2	3	1	3	4	1	3
104	Rua	SeqSNP	2	3	4	3	1	3	2	2	3	2	1	1	1	1	0	2	1	2	2	2	2	2	4
172	Rumba	SeqSNP	1	1	2	1	3	3	3	2	0	2	3	1	2	1	1	1	2	3	2	4	1	4	3
173	Russet Burbank	SeqSNP	1	2	1	2	2	0	3	3	4	0	1	1	3	1	3	3	3	2	3	3	2	2	4
173	Russet Burbank	KASP	1	2	1	2	2	0	2	3	4	0	1	1	3	1	3	3	3	2	3	3	2	2	4
174	Russet Norkotah	SeqSNP	1	1	1	2	0	1	3	4	2	1	2	0	1	2	4	3	3	2	2	2	2	2	3
174	Russet Norkotah	KASP	NA	1	NA	NA	0	NA	NA	4	NA	1	NA	0	NA	NA	4	NA	NA	NA	NA	NA	NA	NA	NA
175	Sabie	SeqSNP	1	2	2	1	3	1	1	3	3	0	2	2	3	2	1	3	3	3	2	1	2	2	3
175	Sabie	KASP	1	NA	2	1	NA	NA	1	3	NA	0	NA	NA	3	NA	NA	3	NA	NA	NA	1	NA	NA	NA
176	Sackfiller	SeqSNP	1	2	2	1	3	1	1	3	3	0	2	2	3	2	1	3	3	3	2	1	2	2	3
176	Sackfiller	KASP	1	NA	2	1	NA	NA	1	3	NA	0	NA	NA	3	NA	NA	NA	NA	NA	NA	1	NA	NA	NA
177	Sandvelder	SeqSNP	3	2	0	3	1	1	1	1	2	1	1	1	3	2	2	2	2	2	0	1	2	2	4
177	Sandvelder	KASP	3	2	0	3	1	1	1	1	2	1	1	1	3	2	2	2	2	2	0	1	2	2	4
178	Santé	SeqSNP	2	0	2	2	2	1	3	1	3	3	1	2	1	1	3	2	3	3	3	4	3	1	2
179	Sarpo Mira	SeqSNP	1	2	3	1	3	3	1	2	3	2	2	2	3	2	2	4	2	3	3	3	1	1	3
180	Shepody	SeqSNP	2	4	2	0	1	0	3	0	0	2	2	1	1	2	3	1	3	1	0	2	0	1	3
180	Shepody	KASP	2	4	2	0	1	0	2	0	0	2	2	1	1	2	3	1	3	1	0	2	0	1	3
181	Sifra	SeqSNP	3	3	3	2	2	1	3	3	2	3	3	1	1	1	1	2	2	2	1	4	1	2	1
181	Sifra	KASP	3	3	3	2	2	1	3	3	2	3	3	1	1	1	1	2	2	2	1	4	1	2	1
196	Sound	SeqSNP	2	2	2	2	3	1	3	2	0	3	2	3	1	2	4	0	2	2	3	2	0	3	2
196	Sound	KASP	2	2	2	2	3	1	3	2	0	3	2	3	1	2	4	0	3	2	3	2	0	3	2
182	Spunta	SeqSNP	3	3	3	2	3	1	3	1	1	2	2	2	2	3	1	0	1	1	2	2	3	2	1
182	Spunta	KASP	3	3	3	2	3	1	3	1	1	2	2	2	2	3	1	0	1	1	2	2	3	2	1
8	Taisiya	SeqSNP	3	2	3	3	3	2	3	2	3	3	3	2	2	2	2	0	2	2	0	2	2	1	2
8	Taisiya	KASP	3	2	3	3	3	2	3	2	3	3	3	2	2	2	2	0	2	2	0	2	2	1	2
15	Taurus	SeqSNP	0	0	2	4	3	2	3	2	3	2	2	2	3	1	1	0	2	2	1	2	2	3	2
15	Taurus	KASP	0	0	2	4	3	2	3	2	3	2	2	2	3	1	1	0	2	2	1	2	2	3	2
183	Teton Russet	SeqSNP	2	1	2	2	1	2	3	3	4	3	2	1	1	3	4	1	3	2	3	4	2	3	4
183	Teton Russet	KASP	2	1	2	2	1	2	3	3	4	3	2	1	1	3	4	1	3	2	3	4	2	3	4
184	Toronto	SeqSNP	1	1	3	3	2	2	3	2	2	2	2	1	2	0	4	3	3	2	3	1	2	3	1
185	Ulster Chief	SeqSNP	1	1	1	2	3	2	3	2	2	2	4	1	0	1	1	2	1	2	0	2	1	4	1
186	Umatilla Rus	SeqSNP	2	0	1	2	2	0	3	2	2	2	3	0	2	0	3	1	2	2	1	3	2	2	2
29	Up to Date 2007	SeqSNP	1	2	2	1	3	1	1	3	3	0	2	2	3	2	1	3	3	3	2	1	2	2	3
29	Up to Date 2007	KASP	1	2	2	1	3	0	1	3	3	0	2	2	3	2	1	3	3	3	2	1	2	2	3
29_d	Up to Date 2007_d	SeqSNP	1	2	2	1	3	1	1	3	3	0	2	2	3	2	1	3	3	3	2	1	2	2	3
29_d	Up to Date 2007_d	KASP	1	2	2	1	3	0	1	NA	3	0	2	2	3	2	1	3	3	3	2	1	2	2	3
187	Up to Date 2012	SeqSNP	1	2	2	1	3	1	1	3	3	0	2	2	3	2	1	3	3	3	2	1	2	2	3
187	Up to Date 2012	KASP	1	NA	2	1	3	0	1	3	3	0	2	2	3	2	1	3	3	3	2	1	2	2	3
187_d	Up to Date 2012_d	SeqSNP	1	2	2	1	3	1	1	3	3	0	2	2	3	2	1	3	3	3	2	1	2	2	3
187_d	Up to Date 2012_d	KASP	1	NA	2	1	3	0	1	3	3	0	2	2	3	2	1	3	3	3	2	1	2	2	3
2	Valor	SeqSNP	4	2	3	1	3	2	2	2	4	1	1	4	3	0	2	1	1	2	1	2	1	3	2
2	Valor	KASP	4	2	3	1	3	2	2	2	4	1	1	4	3	0	2	1	1	2	1	2	1	3	2
189	VanDerPlank	SeqSNP	3	2	3	3	2	1	3	2	0	3	2	1	0	1	3	1	3	1	3	4	2	1	2
189	VanDerPlank	KASP	3	2	3	3	2	1	2	2	0	3	3	1	0	1	3	1	3	1	3	4	2	1	2
190	White Lady	SeqSNP	1	2	4	3	3	0	3	0	1	1	1	3	1	2	1	1	4	1	2	2	2	3	3
190	White Lady	KASP	1	NA	4	NA	NA	NA	NA	NA	1	1	NA	3	2	NA	NA	NA	NA	NA	NA	NA	NA	NA	NA
191	Yukon Gold	SeqSNP	2	2	2	1	3	1	2	3	1	3	2	3	3	1	2	1	4	1	3	2	3	3	2

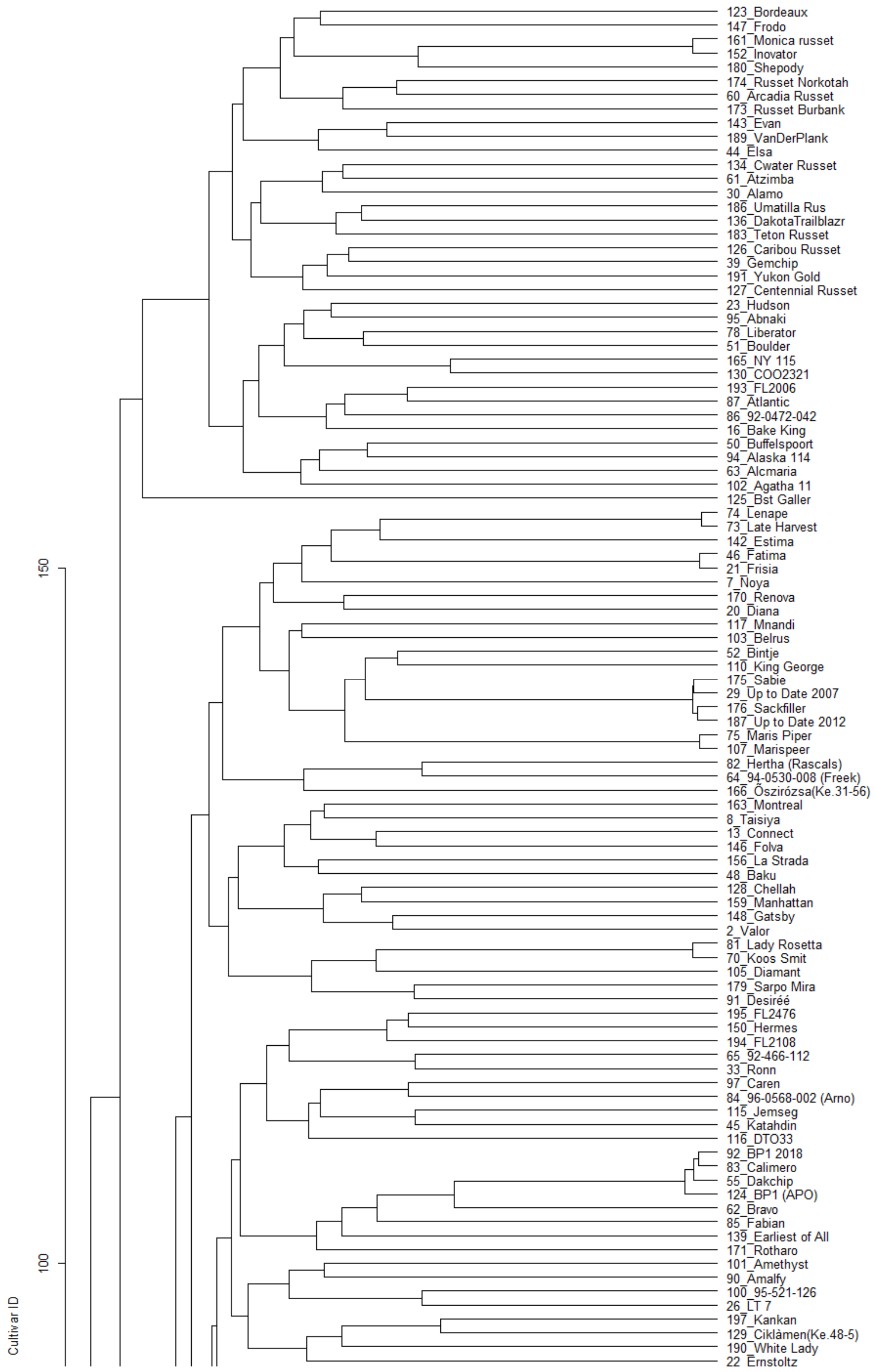
123_Bordeaux
147_Frodo
161_Monica russet
152_Inovator
180_Shepody
174_Russet Norkotah
60_Arcadia Russet
173_Russet Burbank
143_Evan
189_VanDerPlank
44_Elsa
134_Cwater Russet
61_Atzimba
30_Alamo
186_Umatilla Rus
136_DakotaTrailblazr
183_Teton Russet
126_Caribou Russet
39_Gemchip
191_Yukon Gold
127_Centennial Russet
23_Hudson
95_Abnaki
78_Liberator
51_Boulder
165_NY 115
130_COO2321
193_FL2006
87_Atlantic
86_92-0472-042
16_Bake King
50_Buffelspoort
94_Alaska 114
63_Alcmaria
102_Agatha 11
125_Bst Galler
74_Lenape
73_Late Harvest
142_Estima
46_Fatima
21_Frisia
7_Noya
170_Renova
20_Diana
117_Mnandi
103_Belrus
52_Bintje
110_King George
175_Sabie
29_Up to Date 2007
176_Sackfiller
187_Up to Date 2012
75_Maris Piper
107_Marispeer
82_Hertha (Rascals)
64_94-0530-008 (Freek)
166_Öszirózsa(Ke.31-56)
163_Montreal
8_Taisiya
13_Connect
146_Folva
156_La Strada
48_Baku
128_Chellah
159_Manhattan
148_Gatsby
2_Valor
81_Lady Rosetta
70_Koos Smit
105_Diamant
179_Sarpo Mira
91_Desiréé
195_FL2476
150_Hermes
194_FL2108
65_92-466-112
33_Ronn
97_Caren
84_96-0568-002 (Arno)
115_Jemseg
45_Katahdin
116_DTO33
92_BP1 2018
83_Calimero
55_Dakchip
124_BP1 (APO)
62_Bravo
85_Fabian
139_Earliest of All
171_Rotharo
101_Amethyst
90_Amalfy
100_95-521-126
26_LT 7
197_Kankan
129_Ciklámen(Ke.48-5)
190_White Lady
22_Ernstoltz
150
100
Cultivar ID

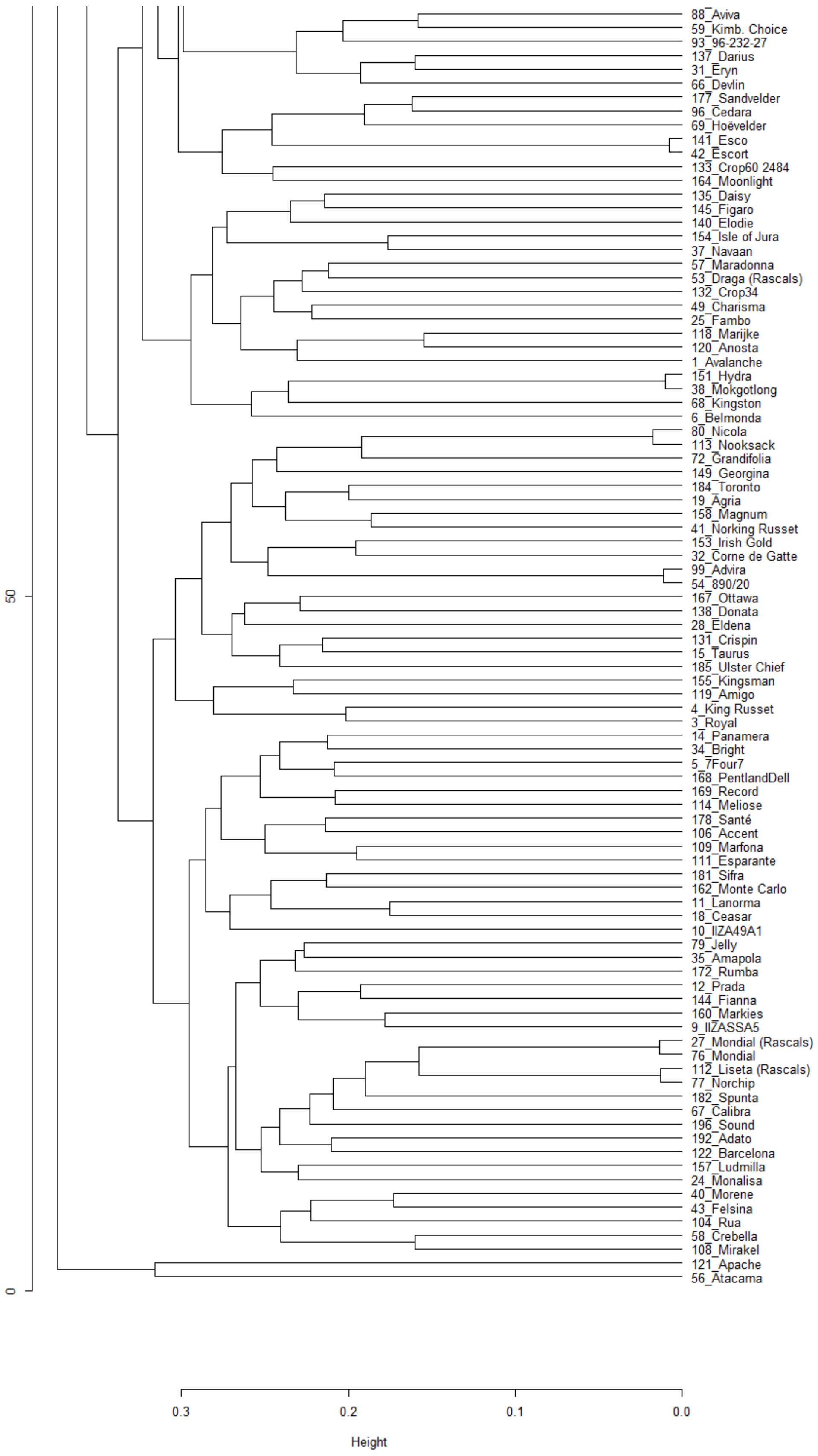

Figure S1 (page 1 and 2). Complete cluster dendrogram of pairwise genetic distances calculated with the Kosman index of 190 cultivars genotyped with SeqSNP at 500 SNP positions.

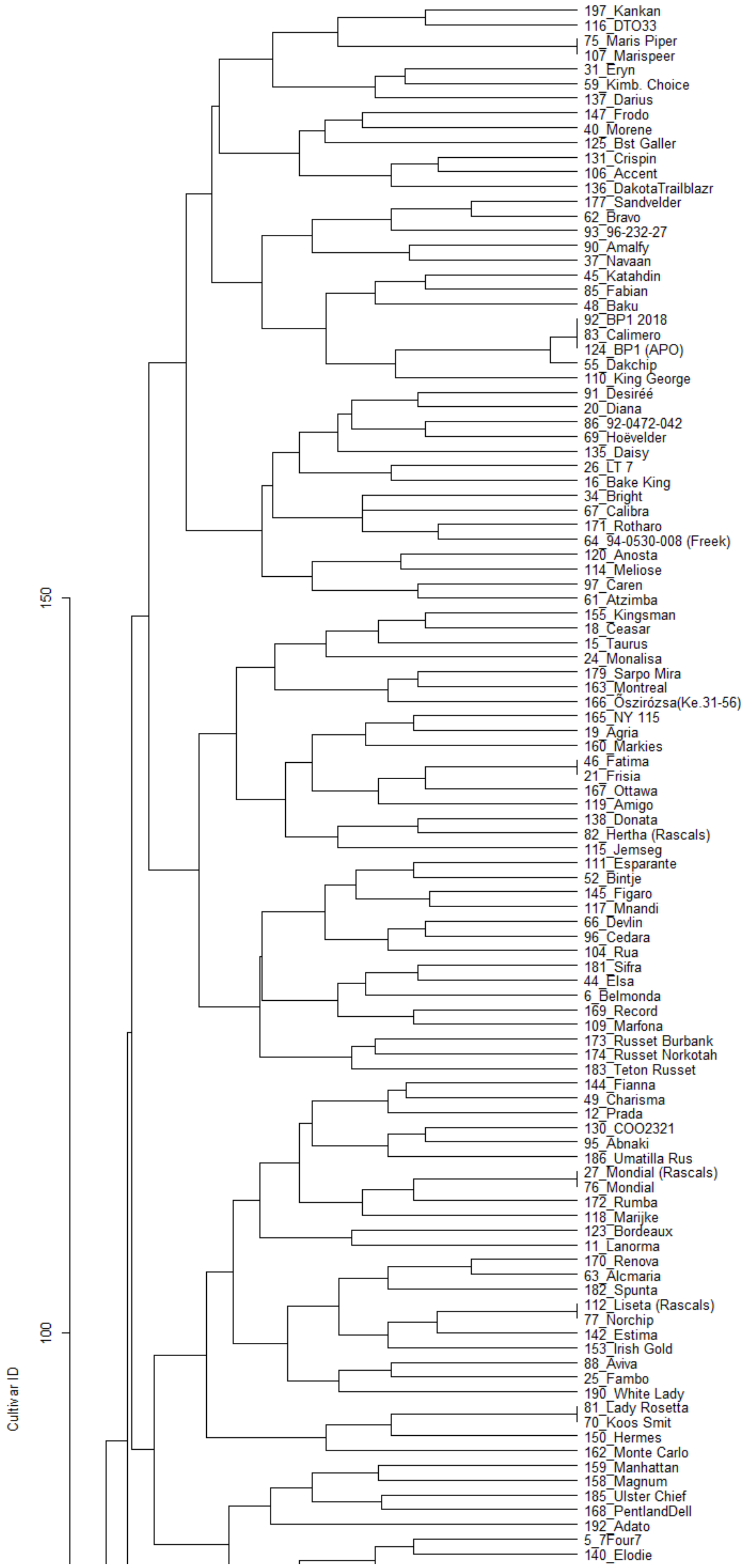
150
100
Cultivar ID
197_Kankan
116_DTO33
75_Maris Piper
107_Marispeer
31_Eryn
59_Kimb. Choice
137_Darius
147_Frodo
40_Morene
125_Bst Galler
131_Crispin
106_Accent
136_DakotaTrailblazr
177_Sandvelder
62_Bravo
93_96-232-27
90_Amalfy
37_Navaan
45_Katahdin
85_Fabian
48_Baku
92_BP1 2018
83_Calimero
124_BP1 (APO)
55_Dakchip
110_King George
91_Desiréé
20_Diana
86_92-0472-042
69_Hoëvelder
135_Daisy
26_LT 7
16_Bake King
34_Bright
67_Calibra
171_Rotharo
64_94-0530-008 (Freek)
120_Anosta
114_Meliose
97_Caren
61_Atzimba
155_Kingsman
18_Ceasar
15_Taurus
24_Monalisa
179_Sarpo Mira
163_Montreal
166_Öszirózsa(Ke.31-56)
165_NY 115
19_Agria
160_Markies
46_Fatima
21_Frisia
167_Ottawa
119_Amigo
138_Donata
82_Hertha (Rascals)
115_Jemseg
111_Esparante
52_Bintje
145_Figaro
117_Mnandi
66_Devlin
96_Cedara
104_Rua
181_Sifra
44_Elsa
6_Belmonda
169_Record
109_Marfona
173_Russet Burbank
174_Russet Norkotah
183_Teton Russet
144_Fianna
49_Charisma
12_Prada
130_COO2321
95_Abnaki
186_Umatilla Rus
27_Mondial (Rascals)
76_Mondial
172_Rumba
118_Marijke
123_Bordeaux
11_Lanorma
170_Renova
63_Alcmaria
182_Spunta
112_Liseta (Rascals)
77_Norchip
142_Estima
153_Irish Gold
88_Aviva
25_Fambo
190_White Lady
81_Lady Rosetta
70_Koos Smit
150_Hermes
162_Monte Carlo
159_Manhattan
158_Magnum
185_Ulster Chief
168_PentlandDell
192_Adato
5_7Four7
140_Elodie

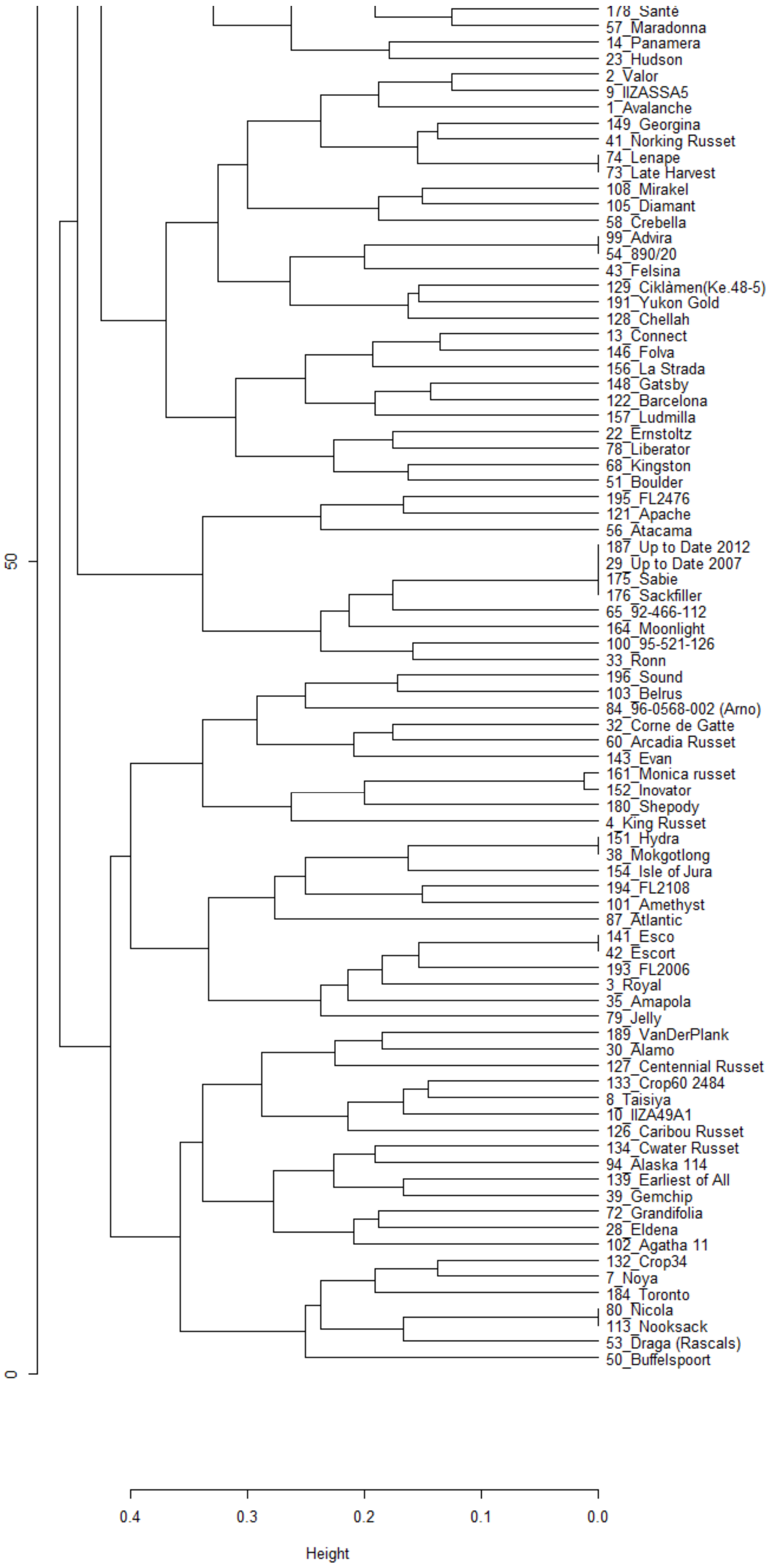

Figure S2 (page 1 and 2). Complete cluster dendrogram of pairwise genetic distances calculated with the Kosman index of 173 cultivars genotyped with SeqSNP at 25 selected SNP positions.

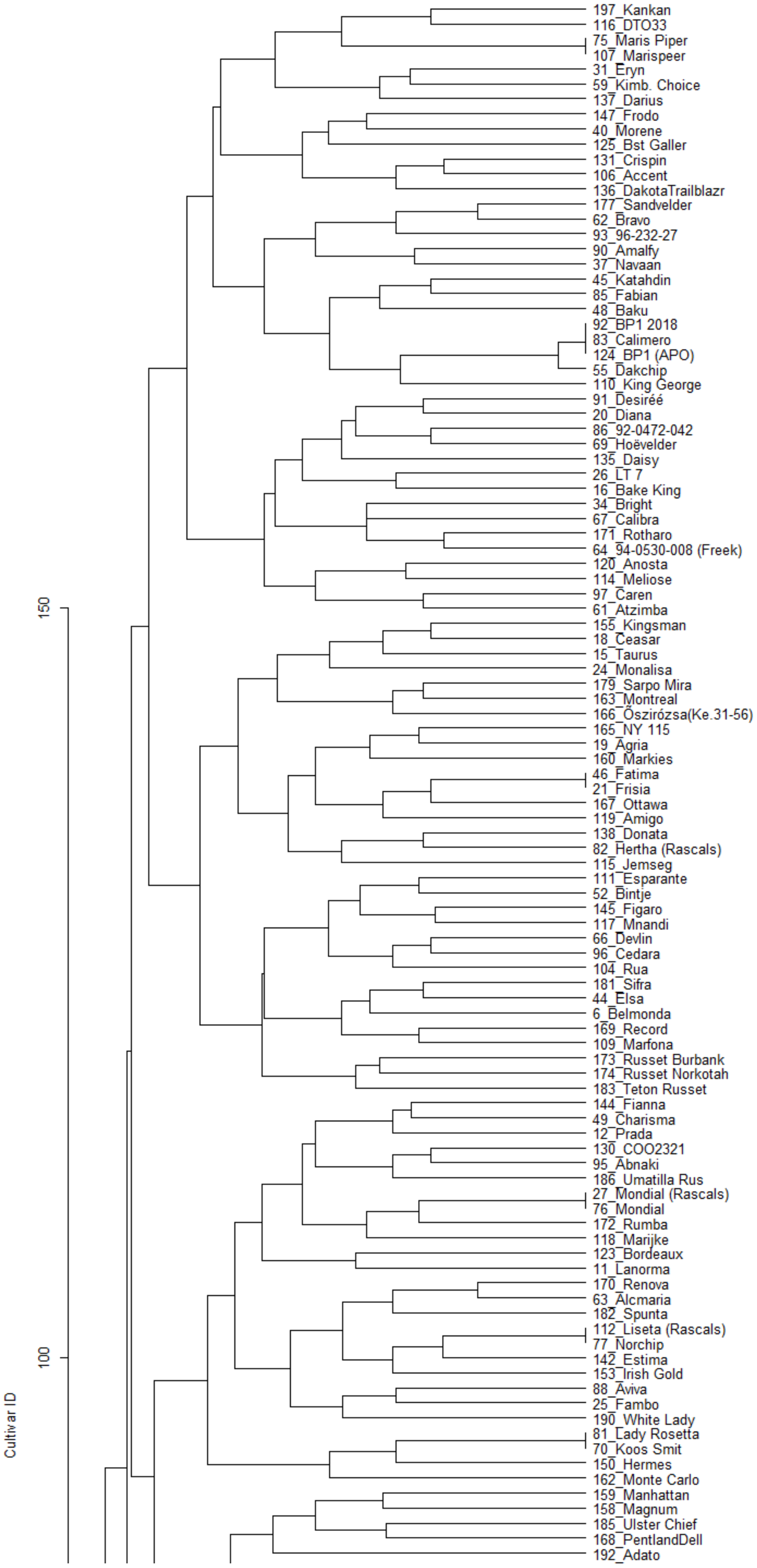
197_Kankan
116_DTO33
75_Maris Piper
107_Marispeer
31_Eryn
59_Kimb. Choice
137_Darius
147_Frodo
40_Morene
125_Bst Galler
131_Crispin
106_Accent
136_DakotaTrailblazr
177_Sandvelder
62_Bravo
93_96-232-27
90_Amalfy
37_Navaan
45_Katahdin
85_Fabian
48_Baku
92_BP1 2018
83_Calimero
124_BP1 (APO)
55_Dakchip
110_King George
91_Desiréé
20_Diana
86_92-0472-042
69_Hoëvelder
135_Daisy
26_LT 7
16_Bake King
34_Bright
67_Calibra
171_Rotharo
64_94-0530-008 (Freek)
120_Anosta
114_Meliose
97_Caren
61_Atzimba
155_Kingsman
18_Ceasar
15_Taurus
24_Monalisa
179_Sarpo Mira
163_Montreal
166_Öszirózsa(Ke.31-56)
165_NY 115
19_Agria
160_Markies
46_Fatima
21_Frisia
167_Ottawa
119_Amigo
138_Donata
82_Hertha (Rascals)
115_Jemseg
111_Esparante
52_Bintje
145_Figaro
117_Mnandi
66_Devlin
96_Cedara
104_Rua
181_Sifra
44_Elsa
6_Belmonda
169_Record
109_Marfona
173_Russet Burbank
174_Russet Norkotah
183_Teton Russet
144_Fianna
49_Charisma
12_Prada
130_COO2321
95_Abnaki
186_Umatilla Rus
27_Mondial (Rascals)
76_Mondial
172_Rumba
118_Marijke
123_Bordeaux
11_Lanorma
170_Renova
63_Alcmaria
182_Spunta
112_Liseta (Rascals)
77_Norchip
142_Estima
153_Irish Gold
88_Aviva
25_Fambo
190_White Lady
81_Lady Rosetta
70_Koos Smit
150_Hermes
162_Monte Carlo
159_Manhattan
158_Magnum
185_Ulster Chief
168_PentlandDell
192_Adato
150
100
Cultivar ID

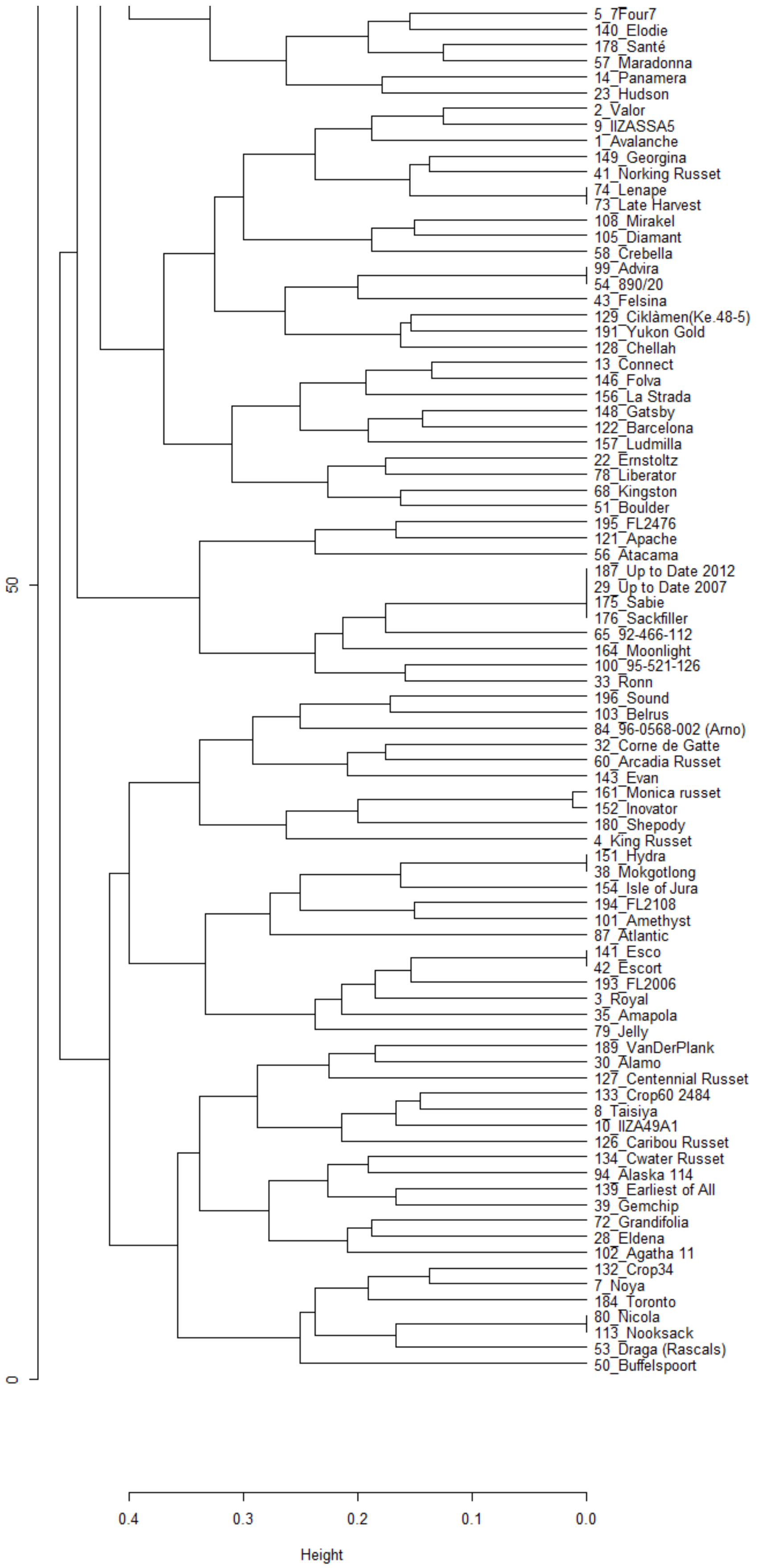

Figure S3 (page 1 and 2). Complete cluster dendrogram of pairwise genetic distances calculated with the Kosman index of 190 cultivars genotyped with SeqSNP at the 21 selected SNP panel positions.

Author Biography

Inge Gazendam has been a researcher at the Agricultural research council (ARC) Vegetable, Industrial and Medicinal plants (VIMP) since 2001. During this time, she has applied a variety of molecular biology, genomics, viromics, virus diagnostics and molecular marker tools to mandated vegetable and indigenous flower plants. She has also performed plant transformation to improve crops for drought and virus tolerance. Inge graduated from the University of Pretoria in 2012 with a Ph.D in Plant biotechnology where she identified various cowpea genes responding to drought stress. Functional characterisation was carried out by transforming Arabidopsis thaliana with a selected drought-induced cowpea gene. Her current interest is in incorporating molecular markers to modernize breeding of various vegetables at ARC-VIMP. She is involved in fingerprinting potato and sweet potato for genetic trueness-to type, and marker-assisted selection for male sterility in onion. She is an alumni of the UC-Davis African plant breeding academy (AfPBA) class IV (Dec 2019).
