# Supplementary figures and images for: Potato Cultivar Identification in South Africa Using a Custom SNP Panel
Source: Plants (Basel). 2022 Jun 10;11(12):1546. doi: 10.3390/plants11121546 (PMC9231109; doi:10.3390/plants11121546)

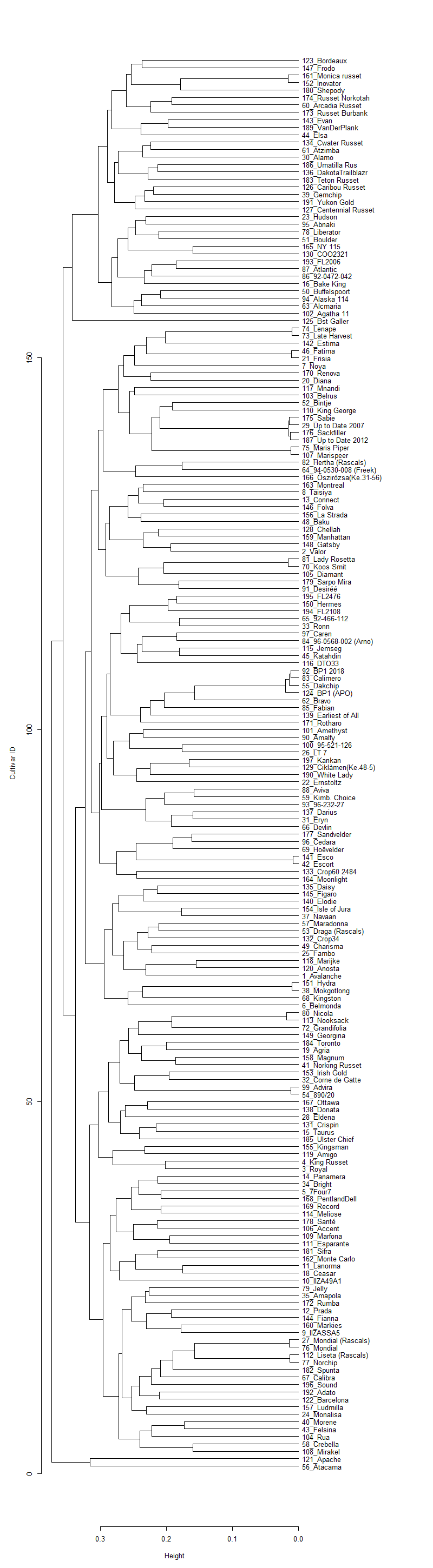

Supplement: Supplementary file 1 [file plants-11-01546-s001.zip › Figure S1_800x2900_SeqSNP 190CV 500 SNP - use.png]

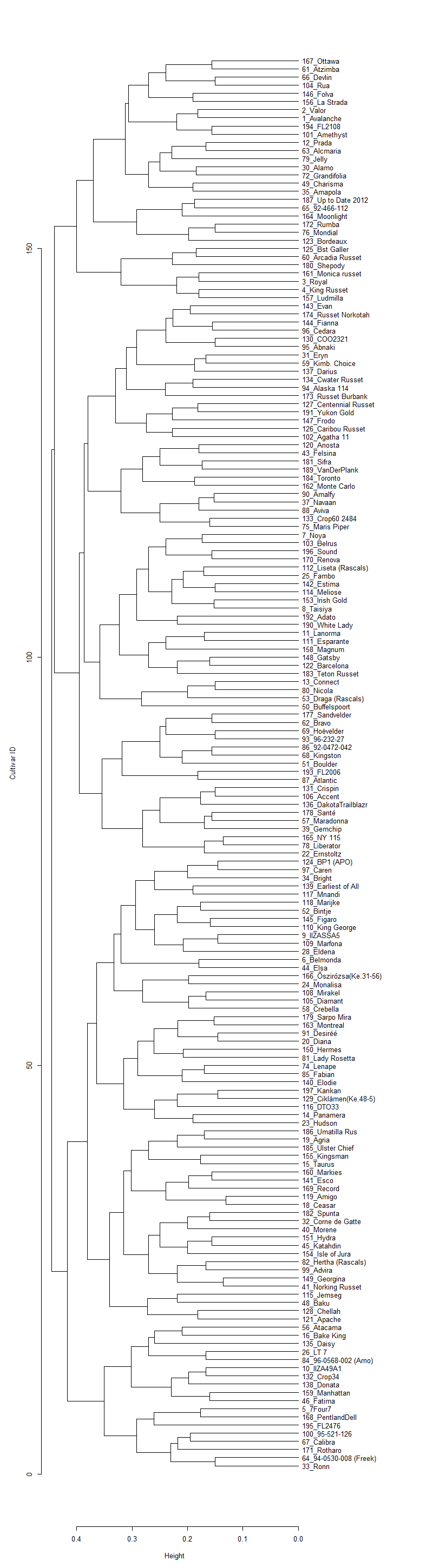

Supplement: Supplementary file 1 [file plants-11-01546-s001.zip › Figure S2_800x2900_SeqSNP 173CV 25 SNP - use.png]

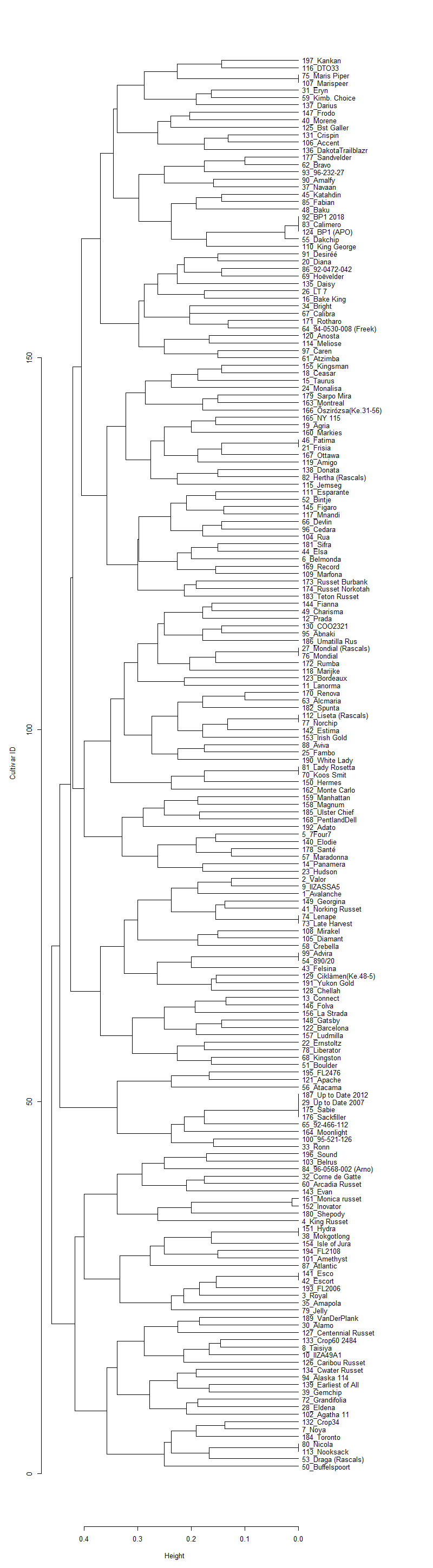

Supplement: Supplementary file 1 [file plants-11-01546-s001.zip › Figure S3_800x2900_SeqSNP 190CV 21 SNP - use.png]
